# Microbiome balance in sputum determined by PCR stratifies COPD exacerbations and shows potential for selective use of antibiotics

**DOI:** 10.1371/journal.pone.0182833

**Published:** 2017-08-25

**Authors:** Koirobi Haldar, Mona Bafadhel, Kelvin Lau, Adam Berg, Brenda Kwambana, Tatiana Kebadze, Mohammadali Yavari Ramsheh, Bethan Barker, Pranabashis Haldar, Sebastian Johnston, Julian M. Ketley, Christopher E. Brightling, Michael R. Barer

**Affiliations:** 1 Department of Infection, Immunity and Inflammation, University of Leicester, Leicester, United Kingdom; 2 Institute for Lung Health, Department of Infection, Immunity & Inflammation, Glenfield Hospital, University of Leicester, Leicester, United Kingdom; 3 Department of Genetics, University of Leicester, Leicester, United Kingdom; 4 Airway Disease Infection Section, National Heart and Lung Institute, Imperial College, London, United Kingdom; 5 Department of Health Sciences, University of Leicester, Leicester, United Kingdom; 6 Department of Clinical Microbiology, University Hospitals of Leicester NHS Trust, Leicester, United Kingdom; National and Kapodistrian University of Athens, GREECE

## Abstract

**Background:**

While a subgroup of patients with exacerbations of chronic obstructive pulmonary disease (COPD) clearly benefit from antibiotics, their identification remains challenging. We hypothesised that selective assessment of the balance between the two dominant bacterial groups (Gammaproteobacteria (G) and Firmicutes (F)) in COPD sputum samples might reveal a subgroup with a bacterial community structure change at exacerbation that was restored to baseline on recovery and potentially reflects effective antibiotic treatment.

**Methods:**

Phylogenetically specific 16S rRNA genes were determined by quantitative real time PCR to derive a G:F ratio in serial sputum samples from 66 extensively-phenotyped COPD exacerbation episodes.

**Results:**

Cluster analysis based on Euclidean distance measures, generated across the 4 visit times (stable and exacerbation day: 0,14 and 42) for the 66 exacerbation episodes, revealed three subgroups designated HG, HF, and GF reflecting predominance or equivalence of the two target bacterial groups. While the other subgroups showed no change at exacerbation, the HG cluster (n = 20) was characterized by G:F ratios that increased significantly at exacerbation and returned to baseline on recovery (p<0.00001); ratios in the HG group also correlated positively with inflammatory markers and negatively with FEV_1_. At exacerbation G:F showed a significant receiver-operator-characteristic curve to identify the HG subgroup (AUC 0.90, p<0.0001).

**Conclusions:**

The G:F ratio at exacerbation can be determined on a timescale compatible with decisions regarding clinical management. We propose that the G:F ratio has potential for use as a biomarker enabling selective use of antibiotics in COPD exacerbations and hence warrants further clinical evaluation.

## Introduction

Chronic obstructive pulmonary disease (COPD) presents a major and increasing disease burden in both well- and poorly-resourced settings [1]. Patients suffer from chronic cough, difficulty breathing and exacerbations that herald progressive deterioration in lung function and wellbeing [2].

Although sputum from patients with COPD is heavily colonized with multiple bacterial species, their importance in the aetiology of exacerbations remains subject to debate with some studies indicating the importance of individual pathogens and others failing to do so [3]. The recently developed capacity to profile microbial communities through sequencing of 16S rRNA gene libraries allows us to analyse relationships between the sputum microbiome and clinical status and test more complex hypotheses concerning the causation of COPD exacerbations than those associated with single pathogens. Indeed the emerging field of microbiomic analysis of clinical specimens increasingly requires microbiologists and clinicians alike to consider the role of changing microbial communities in causing dysbiosis, in this instance meaning a local shift in ecological balance associated with instability in host clinical status distinct from the unitary pathogen hypothesis expressed in the Henle-Koch postulates [4].

We recently described the dynamic changes in the sputum bacterial microbiomes of COPD patients undergoing exacerbation episodes [5]. Overall, and consistent with previous reports, exacerbation was associated with a decrease in community diversity and an increase in the proportion of Proteobacteria. Increases in the proportion of *Moraxella* sequences were present at exacerbation in ~40% of episodes while particular features of the community structure both discriminated between exacerbations designated eosinophilic and bacterial exacerbations and showed dependence on the proportion of *Haemophilus* sequences present. Although these community structure profiles raised interesting possibilities concerning the pathogenesis of exacerbations, such analyses are too resource-intensive to be deployed in day to day clinical practice.

Following on from the observation by Turnbaugh and colleagues [6] that obesity is associated with a disturbance in the ratio of the two major phylogenetic groups in the human gut microbiome we explored and reported in abstract form, preliminary data categorising thirty exacerbation episodes according to the ratio in sputum between the Proteobacteria and the Firmicutes (designated G:F below), determined by 16S gene sequencing [7]. In one of the subgroups this ratio increased at the time of exacerbation and returned to baseline on recovery. This observation led us to hypothesise that the G:F ratio might identify a clinically distinct sub-group in whom a dysbiotic microbiome disturbance at exacerbation might be restored to a more healthy balance though recovery, possibly as a result of effective antibiotic treatment. Accordingly, we have established and investigated a real time PCR assay to directly determine this ratio in sputum samples to test our hypothesis and to explore the possibility that timely G:F assay might have potential to inform clinical practice. Here we report results of G:F assays applied to serial sputum samples representing 66 COPD exacerbation episodes and their correlation with the associated clinical metadata.

## Methods

### Subjects and samples

The subjects and samples studied here were a subset from a prospective observational study undertaken at Glenfield Hospital, Leicester, UK. Study design, patient enrolment criteria, patient demographics, sputum collection and measurements performed have been described previously [8, 9]. Analyses were applied to sequential sputum samples taken at visit times designated: Stable (S) (at least 8 weeks after last exacerbation), exacerbation (Day 0) as defined and treated by Anthonisen criteria [10] and sampled prior to initiation of treatment, and two follow-up samples taken 14 and 42 days later (respectively Day 14 and Day 42). Sixty six exacerbations yielded adequate sputum samples for analysis at all four visit times; thus 264 samples generated by 58 subjects (some subjects had multiple episodes) were analysed here. Clinical metadata and health quality assessments together with sputum and serum for inflammatory biomarkers were collected at all visit times [8, 9]. Quantitative PCR (qPCR) to assess bacterial load and PCR-based virus detection were also performed on these samples. [8, 11].

### DNA extraction

Routine culture tests were performed on the sputum homogenized with an equal volume 0.1% Dithiothreitol (DTT) at Leicester Royal Infirmary (LRI) following the then health protection agency standard operating procedure [12]. Total genomic DNA was extracted from the homogenate using the QIAamp DNA Mini Kit (Qiagen, California, USA) following the "Gram positive bacteria extraction" method, as per the manufacturer’s protocol. Briefly, bacteria harvested from 500μl of homogenized sputum were lysed with 20mg/ml lysozyme and incubated at 37°C for 30 minutes followed by Proteinase K treatment at 55°C for 30 minutes and 95°C for 15 minutes. The remainder of the extraction was done according to the "DNA Extraction from Tissue" method described in the manufacturer’s protocol. This involved adding the bacterial cell lysate to 200μl absolute ethanol to the QIAamp spin column and centrifuging briefly to adsorb the DNA optimally to the column’s silica gel membrane. This was followed by wash steps to remove impurities and finally the DNA was eluted in 200μl of DNAse, RNAse free distilled water. The DNA was stored at -20°C.

### Conventional pathogen detection and Gammaproteobacteria:Firmicutes (G:F) qPCR assay

Diagnostic culture and qPCR for the detection of selected pathogens were performed as described previously [8, 11]. As noted above, inspection of the complex microbiome profiles from a subset of 30 samples from this study led us to consider whether, as suggested by the work of Turnbaugh and colleagues [6], the balance between the major phylogenetic groups might be more readily linked to clinical status. Review of the ratios between the two dominant bacterial groups (Gammaroteobacteria and Firmicutes), comprising >80% of the 16S gene sequences detected, indicated a subgroup with changes at exacerbation that restored to baseline during recovery [7]. To explore this in a larger sample group a Gammaprobacteria and Firmicutes qPCR was developed. Primers targeting the Gammaproteobacteria were used as this group was overwhelmingly dominant within our Proteobacteria sequences and primers targeting the whole phylum were not available (see Table A in S1 File).

For the G:F ratio assay, Gammaproteobacteria and Firmicutes were targeted respectively with ϒproteo871F[13]/ϒproteo1202R[14] and Firm928F/Firm1040R [14] primers. These primer pairs were chosen from published sets following *in silico* checks against the ribosomal database project (RDP; http://rdp.cme.msu.edu/) to confirm coverage and specificity (Table A in S1 File). Sensitivity and specificity of the selected primers for the target groups was confirmed by performing qPCR assays in varying proportions of pure culture mixtures of *Streptococcus pneumoniae* and *Haemophilus influenzae* (Figure A in S1 File).

Extracted DNA samples from cultures of *H*. *influenzae* and *S*. *pneumoniae* (107–10^2^ genome/μl), were used as standards for the Gammproteobacteria:Firmicutes qPCR assay. Reactions for both assays were prepared with 12.5μl 2x Absolute qPCR SYBR Green PCR mix, 9.5μl water with 1μl of each modified ϒproteo871F (5' TAAGTHGACCGCCTGGGGAGT 3') [13] and ϒproteo1202R (5' CGTAAGGGCCATGATG 3') [14] primers at a final concentration of 200nM for the Proteobacteria assay and 1μl of each Firm928F (5’ TGAAACTYAAAGGAATTGACG 3')[14]and Firm1040R (5’ ACCATGCACCACCTGTC 3’)[14] at a final concentration of 150nM for the Firmicutes assay. 1μl of template DNA was added making the final reaction volume 25μl. Samples were quantified in duplicates simultaneously for both bacterial groups on a Rotor-Gene 6000 (Corbett Life Sciences, UK). Cycling conditions were 95°C for 15 minutes followed by 40 cycles of 95°C for 20s, 60°C for 30s, 72°C for 20s, and data acquisition at 78°C for 20s. Finally melt (T_m_) curve analysis was performed from 65°C to 99°C to determine the specificity of the amplicons. Samples from qPCR runs with correlation coefficients (R^2^) > 0.98, amplification efficiencies >0.7, less than 1 Ct difference between duplicates and melt temperatures within ± 0.5°C of each other were accepted [15], discrepant assays were repeated.

There was a good correlation (r^2^ = 0.73. p<0.0001; Figure B in S1 File) between qPCR-determined ratios and ratios derived from our earlier bacterial community analyses by Roche 454 sequencing [7].

### Statistical analysis

Univariate statistical analyses were performed using GraphPad Prism (Version 5, San Diego, CA). Parametric and nonparametric data are presented as mean (SEM) and median (interquartile range) respectively. For continuous parametric data, unpaired student t-tests and one way analysis of variance (ANOVA) were performed for between-group comparison of two or greater than two groups respectively. For within-group longitudinal comparisons, the paired t-test (comparison between two visits) and repeated measures analysis of variance (RM-ANOVA; comparison across greater than two visits) were used. For ANOVA and RM-ANOVA, Tukey's HSD procedure was used to determine statistical significance of pair-wise comparisons. Equivalent non-parametric statistical tests were performed for non-parametric data.

The heatmap2 function in R software version 3.0.2 (http://www.R-project.org) was used to perform hierarchical clustering (HC). Clusters were defined according to the pattern of change across visits within each episode. The Euclidean distance matrix was constructed using the sum of pairwise comparison of G:F ratio at each of the four visit times between exacerbation episodes (Formula A in S1 File). This technique retains the integrity of the relationship between samples of the same episode.

### Ethics approval

The study was conducted in accordance with the amended Declaration of Helsinki and was approved by the Leicestershire, Northamptonshire and Rutland ethics committee (07/H0406/157). All subjects gave written informed consent.

## Results

Clinical characteristics of the 58 subjects are summarized in Table 1. Disease severity ranged from GOLD stage 2 to 4 and both current and ex-smokers were included.

**Table 1:** Patient characteristics.

Patient characteristics (n = 58)	
Age	68.8 (1.2)
Male, n (%)	46(79)
Current smokers, n (%)	23 (39)
pack year history	44 (3)
Exacerbation over preceding year	3 (0.3)
BMI, kg/m2	26.51(0.62)
FEV_1_, L	1.3(0.1)
FEV_1_, % predicted	50 (2.5)
FEV_1_/FVC %	47 (2)
ICS(BDP equivalent)dose, mcg	1423 (87)
SGRQ score	51.45 (2.39)
Blood CRP, mg/L~	3 (3–9)
Sputum neutrophil %	69.8 (3.2)
GOLD 1+2	51%
GOLD 3	33%
GOLD 4	16%

Data expressed as mean (SEM) unless otherwise stated; ~median (IQR); FEV_1_, % predicted = Spirometry recorded post bronchodilator; BMI = body mass index; FEV_1_ = forced expiratory volume in 1 second; FVC = forced vital capacity; BDP = beclometasone dipropionate; SGRQ = St George’s Respiratory Questionnaire; CRP = C reactive protein; ICS = inhaled corticosteroid.

### The G:F ratio reveals three main subgroups

The G:F qPCR method was successfully applied to all 264 available samples.

**Fig 1:**
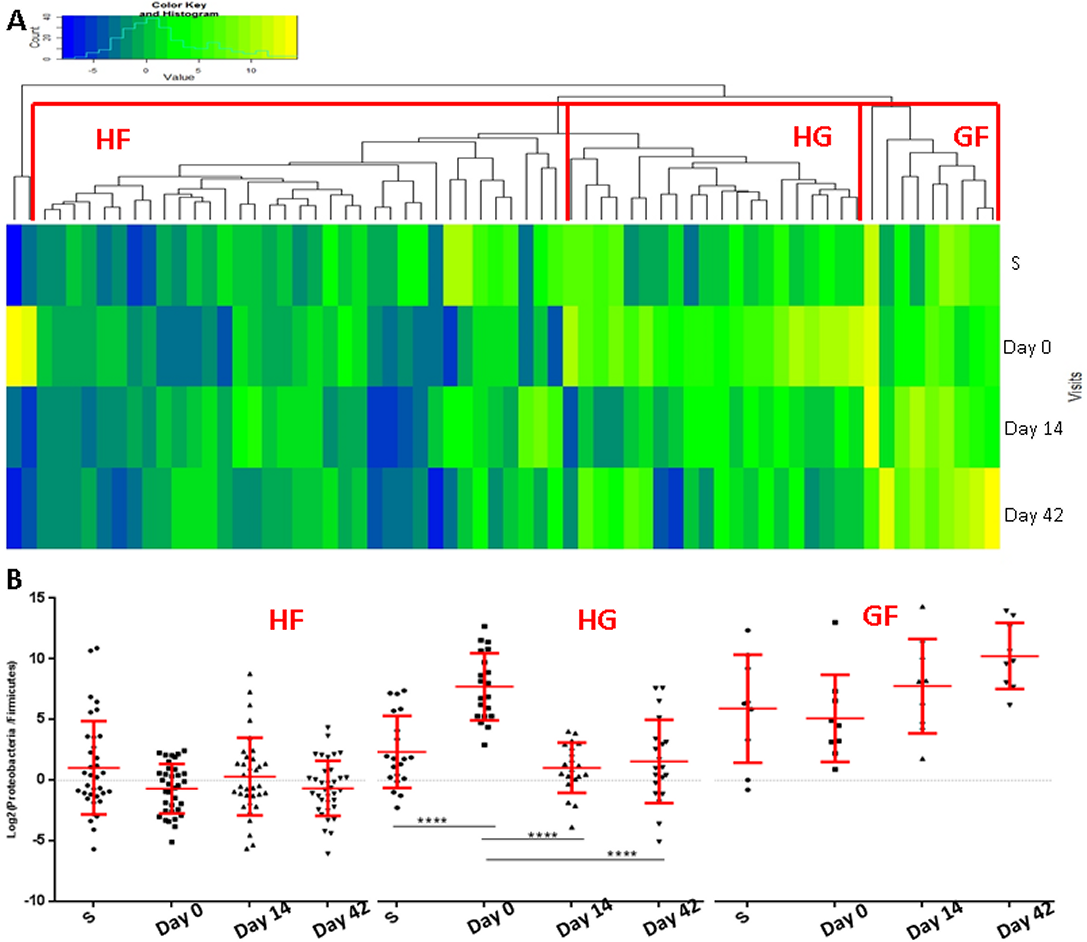
Cluster analysis of qPCR-determined G:F ratios reveals three subgroups with different patterns of change through exacerbation. **(A)** Heatmap representing the clustering of exacerbation episodes based on G:F ratio pattern across the four visit times. Blue shows Firmicute dominance and yellow Gammaproteobacterial dominance. **(B)** Changes in G:F across visit times. Mean ±SD. Points represent the individual sample G:F ratios.(****) p<0.0001. HF = High Firmicutes, HG = High Gammaproteobacteria and GF = Balanced G:F.

To determine whether G:F ratios identified distinct exacerbation subgroups, we performed unsupervised hierarchical clustering of the ratios in their four visit sample sets representing the 66 captured exacerbations. We emphasise that this approach took into account the results at all four visit times for each of the exacerbation events analysed and identified their relatedness to every other exacerbation event by pairwise comparison. The model identified three major clusters incorporating 64 of the events. These were designated HG (High Gammaproteobacteria, n = 20), HF (High Firmicutes, n = 35) and GF (Balanced G:F, n = 9) (Fig 1A). Two outlier events showed a Firmicute dominated stable ratio and, like the HG group, a shift towards the Gammaproteobacteria at exacerbation followed by a return to the stable pattern during recovery.

A significant increase in G:F ratio (p<0.00001) was apparent at exacerbation in the HG cluster and this returned to stable visit levels in the follow up and recovery visits (p<0.00001; Fig 1B). Moreover, the shift in the G:F ratio observed at exacerbation in the HG cluster was attributable to concomitant significant (p≤0.008) increases and decreases in PCR-determined Gammaproteobacteria and Firmicute signals respectively (Figure A in S2 File). The HF cluster was characterized by a relatively stable G:F ratio at all visits with little change through exacerbations. In contrast, the GF cluster showed a trend to a higher G:F ratio at recovery and follow-up. Between-cluster differences in G:F ratio were greatest at exacerbation (HG>GF>HF; p<0.0001).

Comparison of the baseline characteristics in the three clusters (Table 2) revealed that subjects in the HF cluster had had more exacerbations in the preceding year (4 vs 2.2; ANOVA p = 0.01) and a significantly higher St Georges Respiratory Questionnaire [16] total symptom score (p = 0.038). No significant associations were found with GOLD stage, gender, smoking status, or treatment given.

**Table 2:** Comparison of patient characteristics between G:F clusters.

		HF (n = 30)	HG (n = 17)	GF (n = 9)	p
	Age	69.7 (1.7)	67.9 (2.6)	68.1 (1.4)	0.78
	Male, n(%)	24 (80%)	13 (76%)	8 (88%)	0.74
	Current smokers, n(%)	13 (43%)	6 (35%)	3 (33%)	0.8
	pack year history	43 (5)	46 (6)	46 (10)	0.93
	Exacerbation over preceding year	4 (0.5)	2.2 (0.4)	2.0 (0.5)	**0.01**
	BMI, kg/m2	26.6(0.9)	27 (1.0)	25.9 (1.4)	0.86
	ICS (BDP equivalent) dose, mcg	1489(122)	1257(163)	1533(185)	0.13
	SGRQ score	57.1 (2.6)	45.2 (5.4)	43.7 (5.6)	**0.038**
Disease severity	GOLD 1+2	15 (50%)	11 (65%)	4 (44%)	0.84
	GOLD 3	10 (33%)	4 (23%)	3 (33%)	
	GOLD 4	5 (17%)	2 (12%)	2 (22%)	
*Treatment at exacerbation	Antibiotics and steroids	20 (57%)	11 (55%)	4 (45%)	0.12
	Steroids only	2 (6%)	1 (5%)	3 (33%)	
	Antibiotics only	13 (37%)	8 (40%)	2 (22%)	

Data are presented for the 56 subjects with 64 exacerbation episodes that were assigned to one of the three major clusters.

* Includes all 64 exacerbation episodes. Data presented as mean (SEM); n = number of subjects in each cluster; BMI = body mass index; BDP = beclomethasone dipropionate; SGRQ = St George’s Respiratory Questionnaire.

### The HG cluster was significantly associated with dynamic changes in inflammatory markers and FEV_1_ over the time course of an exacerbation episode

The increase in the G:F ratio exclusive to the HG cluster was strongly associated with mean serum CRP levels across the sample visits (Day 0> Day 42> Day 14>Stable, p = 0.0002). When the analysis was confined to episodes for which we had complete sets of metadata (Table 3), the HG cluster demonstrated a significant change across visits in CRP (p = 0.004), sputum IL-1β (p = 0.03) and sputum neutrophil % (p = 0.01) that was not seen in the other groups. The changes in the HG group were characterised by a significant increase in these biomarkers at exacerbation (Day 0) and recovery to stable levels with treatment.

**Table 3:** Comparison of inflammatory markers between the three clusters.

		Stable	Day 0	Day 14	Day 42	p
CRP	HF (26)	5 (2.5–10)	9.5 (2.5–23.25)	5.5 (2.5–10.8)	2.5 (2.5–10)	0.524
	HG (15)	2.5 (2.5–2.5)	13 (6–31)	2.5 (2.5–8)	2.5 (2.5–10)	**0.004**
	GF (4)	7.8 (2.5–14)	8 (3.4–16)	2.5(2.5–35.9)	9 (3.9–54.3)	0.58
%Neutrophil	HF (20)	56.1 ± 5.6	69.7 ± 5.8	67.8 ± 5.4	54.3 ± 4.9	**0.04**
	HG (14)	65 ± 6.4	85.5 ± 5.9	81.9 ± 3.8	70.6 ± 7.3	**0.01**
	GF (6)	87 ± 4.7	88.8 ± 5	88.5 ± 6.4	87.1 ± 6.1	0.94
IL-1β	HF (13)	89 (21–232)	50 (29–398)			0.5
	HG (6)	96 (25–466)	719 (114–4722)			**0.03**
	GF (2)	1110 (31–14862)	492 (121–11012)			n to small

Data presented as mean±SEM values for parametric data and median (IQR) for non parameric data p values for differences across all 4 visits in matched samples in individual G:Fcluster. % Neutrophils and IL-1β were determined in sputum.

**Fig 2:**
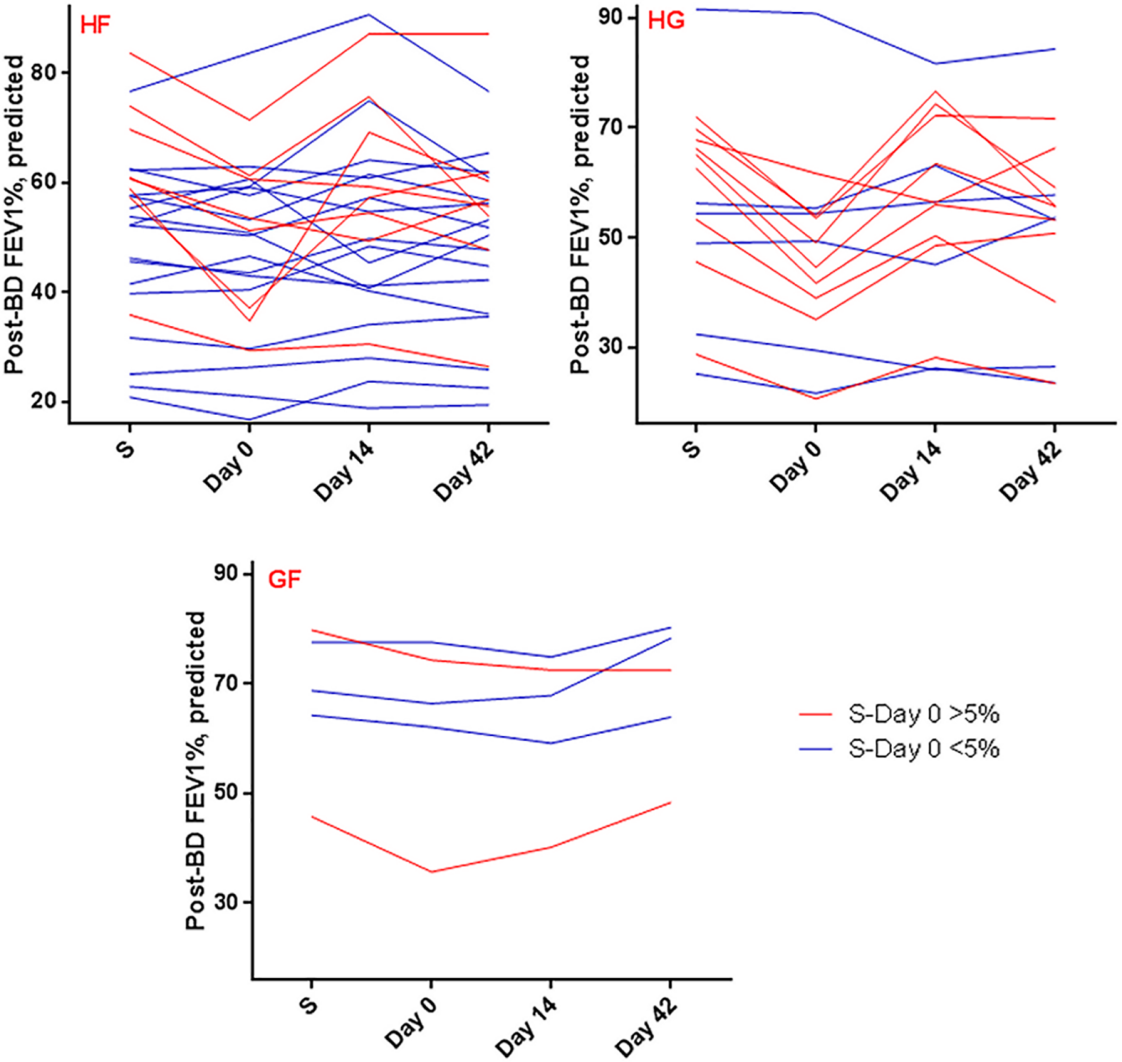
Comparison of post-bronchodilator FEV_1_% across visit times in individual clusters shows significant decline in lung function at exacerbation in the HG cluster. Each line connects individual an exacerbation episode across the 4 visit times. Episodes showing decline in Post-BD FEV_1_ between stable and Day 0 of >5% are represented in red and those with lesser declines at this visit are shown in blue. Note the preponderance of red lines in the HG cluster and blue lines in the HF cluster.

The HG cluster also showed a significant (p = 0.0014) decline in post bronchodilator (BD) FEV_1_%, predicted at exacerbation that recovered to stable visit values with treatment (Mean Diff ± SE = 9 ± 2 (stable), 8 ± 2.8 (Day 14) and 5 ± 1.8 (Day42)). In contrast, post BD FEV_1_ in the other clusters showed < 5% mean decline at exacerbation (Fig 2).

### G:F analysis at exacerbation (day 0) identifies HG group membership better than conventional culture or pathogen directed PCR

The HG subgroup identified by G:F analysis was characterised by correlated changes in microbiome, inflammatory markers and respiratory function, whereas the microbiome was dissociated from these changes in the HF and GF groups. Thus, judged by changes in the microbiome, antibiotic therapy appeared to be effective in the HG group but not relevant to recovery in the other groups. On this basis recognition of HG cluster membership at the time of exacerbation may provide a means of selecting patients most likely to benefit from antimicrobial treatment. We therefore tested the capacity of analyses performed at exacerbation (Day 0) to identify HG cluster membership.

**Fig 3:**
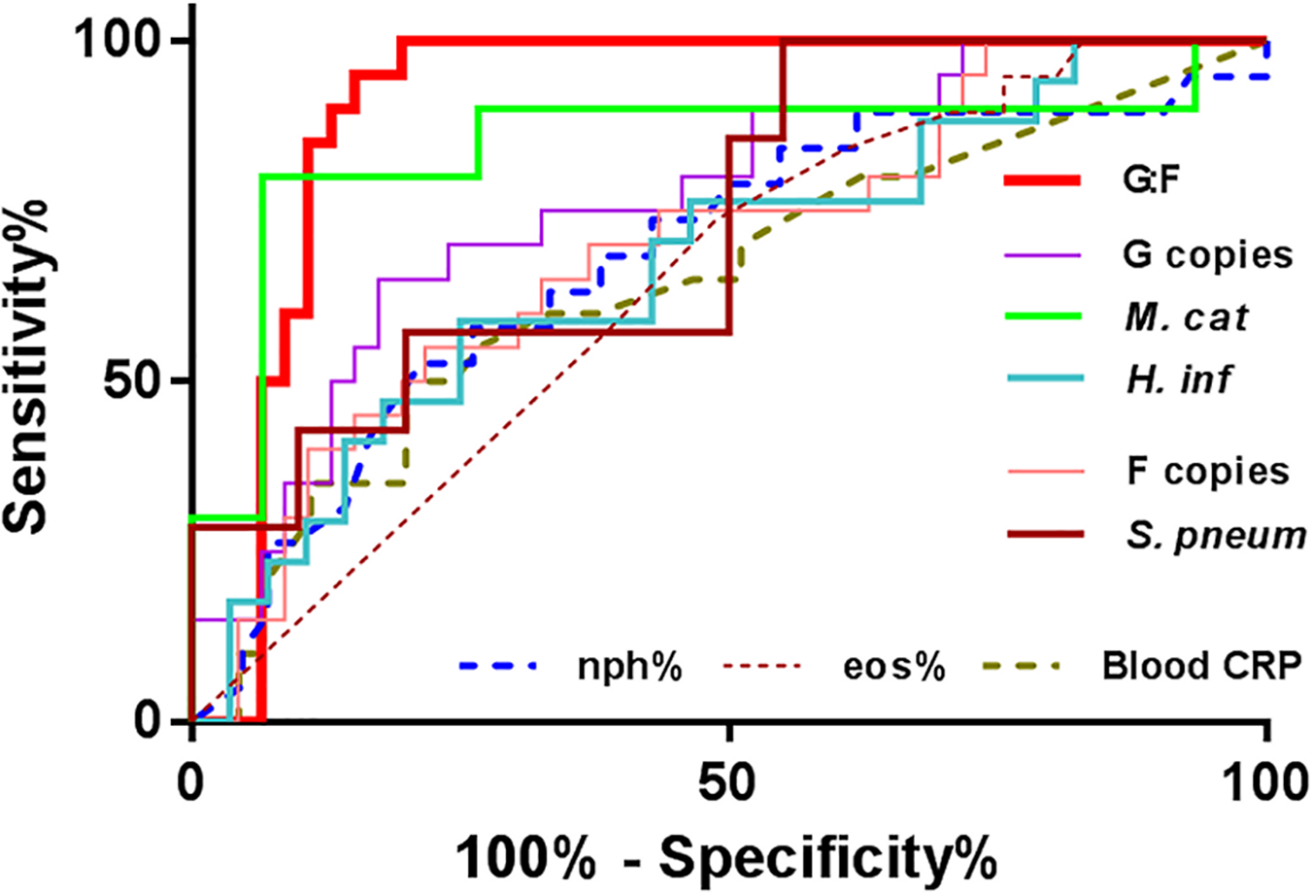
A single G:F ratio assay at exacerbation identifies membership of the HG cluster. Abbreviations G—Gammaproteobacteria; *M*. *cat–M*. *catarrhalis*; *H*. *inf–H*. *influenzae*; F–Firmicutes; *S*. *pneum–S*. *pneumoniae*; nph—neutrophils; eos–eosinophils.

ROC analyses of CRP, granulocyte subsets and G:F ratio are shown in Fig 3. PCR-determined sputum G:F ratio proved the best identifier of the HG group (AUC 0.90, p<0.0001) and a threshold value of 2.67 detected the cluster with 100% sensitivity and 80% specificity. We explored combinations of parameters for their predictive performance. For Il-1 beta, such analysis was limited by an incomplete dataset. Combinations that included CRP and the neutrophil count with G:F performed less well than G:F alone. Culture and qPCR positivity (>10^5^ genome/ml) for specific organisms showed good sensitivity and negative predictive value (Table 4), however they showed weaker specificity and positive predictive values than the G:F assay for identifying the HG group.

**Table 4:** Exacerbation G:F ratio identifies HG membership better than culture and qPCR.

	Sensit-ivity	Specificity	Positive predictive value	Negative predictive value	P value
Culture	0.75	0.73	0.56	0.86	0.008
*qPCR	0.95	0.3	0.38	0.93	0.047
≠G:F ratio	1	0.8	0.69	1	<0.0001

Culture and qPCR (* >10 ^5 genome/ml) positives at exacerbation for *M*. *catarrhalis*, *H*. *influenzae and S*. *pneumoniae* to identify the HG cluster membership.

≠G:F values are determined at threshold of ≥2.67 from ROC analysis.

PCR-based viral analysis was done in our COPD subjects (8). Viruses were detected in 15 out of 66 exacerbations but there was no significant association between virus positivity in the HG cluster (23%) compared to the non HG-associated events (21%) (Table E in S2 File)

## Discussion

Using an assay that assesses the balance between the two numerically dominant bacterial groups in COPD sputum samples, we have identified a subgroup of exacerbation events in which this balance is disturbed at exacerbation and returns to baseline with clinical recovery. Critically, in this high Gammaproteobacteria (HG) subgroup, the G:F ratio correlates well with key acute inflammatory markers and contemporaneous respiratory status (FEV_1_). We propose that acute G:F determinations should be evaluated in further prospective clinical studies as a possible means of differentiating COPD exacerbation events that are more likely to benefit from antibiotic treatment from those where the outcome would not be improved by their use.

Our G:F ratio results were subjected to unbiased cluster analysis, taking account of the four visit time results for all 66 exacerbations studied. This approach identified the three major patterns, HG, HF and GF that were associated with different patterns of G:F changes through the course of each episode. In particular, the HG cluster showed an increase at exacerbation which was restored to baseline during recovery. In contrast, the HF cluster showed no significant change in ratio over the four visit times, while the GF cluster showed a disturbance that was apparent at the follow up and recovery visit times, perhaps reflecting the effects of therapy. Thus at exacerbation the HG subgroup showed a shift in the ratio between the dominant bacterial groups present and this balance was unperturbed at that time in the other clusters.

Interestingly, on comparison of baseline clinical characteristics among the three G:F clusters, the frequency of exacerbations was (~2-fold) higher in the HF compared to the HG and GF clusters; this observation accords with the reported increased frequency of exacerbations associated with *Veilonella* (Firmicutes) rich populations [17]. This could also reflect response to antibiotics in the HG group and lack thereof on the HF group in keeping with previous reports of antibiotic-associated suppression of Proteobacteria [18, 19]. The HF group also showed higher baseline (SGRQ) symptom score that is likely to lower the threshold for identifying exacerbations in this group. Lack of change in G:F through exacerbations might indicate a non-bacterial exacerbation aetiology.

Antibiotic stewardship limiting unnecessary prescription is currently recognised to be of overriding importance in limiting bacterial resistance [20]. Heterogeneity in exacerbation aetiology, lack of generally accepted biomarkers for guiding treatment, and a “precautionary approach” to patients with heavily colonised lower airways may underpin excessive use of antibiotics in COPD. We suggest that both the pattern of G:F ratios and the association with inflammatory and FEV_1_ changes in the HG group and the lack thereof in the other groups, indicate that antibiotics may not have contributed to symptomatic relief in the non HG exacerbations.

Notably, we have shown that the G:F ratio at exacerbation can identify the HG group membership. This can be generated on a timescale compatible with decisions on clinical management and will allow its potential as a biomarker informing decisions on whether or not to treat with antibiotics to be tested. Although the culture results were frequently positive for a recognised pathogen in the HG group, we emphasise that we have previously shown in recognised pathogens, including *H*. *influenzae*, *M*. *catarrhalis* and *S*. *pneumoniae*, that their culture positivity or qPCR signal patterns do not show any consistent correlation with clinical symptoms even in a subgroup of patients [8]; moreover, there were significant levels of culture positivity in the other G:F defined subgroups (27%), reducing the specificity of positive culture for the HG subgroup (c.f. Table 4).

Selection of the two dominant groups of the sputum microbiome for G:F ratio analysis here also reflected the emerging evidence that the balance between phylogenetic groups is linked to pathophysiological status and processes. In particular, analysis of the relative abundance of Firmicutes and Bacteroidetes in the gut microbiome has proven informative in studying obesity and can be linked to plausible biochemical mechanisms through metagenomics [6]. We speculate that mechanistic explanations relating to dominance of Gammaproteobacteria may also be found here.

The key limitation of our study was the relatively low number of subjects, particularly in the HG subgroup (n = 20), and absence of comparative antimicrobial resistance data between the groups. In addition the inclusion of multiple exacerbations from the same subject also requires caution. Our clustering method associates exacerbations rather than subjects. Nonetheless, the clinical correlations we have found are striking and worthy of further exploration.

Concerning our molecular methodologies, questions can be raised regarding our DNA extraction method and G:F primer selection. In the former case, a bead beating method is preferred by some authors to detect Gram positive bacteria [21] while others have used enzymatic methods similar to ours [22, 23] and have shown similar core bacterial constituents to those obtained by bead beating [24]. Although the primer pairs chosen showed better coverage insilico compared to other published primers, limitations in their coverage remain (Table A in S1 File). Thus, although GF ratios from qPCR correlated well with the sequencing based ratio (see Figure B in S1 File), the phylogenetic specificity of our assay is not certain. This issue is compounded by disagreements regarding the composition of phylogenetic groups. At one level, given the clinical correlations seen here, this does not matter, however, the uncertainty does undermine our ability to identify contributions attributable to specific organisms. Clearly if the associations we report here are substantiated, it will be desirable to determine the underlying mechanisms. It is our opinion that these will be better investigated by metagenomic and/or metatranscriptomic methods than by more detailed analysis of the community composition. Thus, while its shortcomings make the G:F ratio somewhat empirical in nature, its application may open up new opportunities to understand the roles of bacteria in COPD exacerbations.

Finally, the suggestion that the HG group could selectively benefit from antibiotics rests on the correlated patterns of G:F, inflammatory markers and FEV_1_ results and the evidence is therefore only circumstantial. Prospective studies stratifying treatment on the basis of G:F cluster membership will provide the results needed for validation of our proposals. We conclude that the G:F PCR applied to sputum could readily be deployed in diagnostic laboratories and merits further investigation as a biomarker with potential to enable more selective and appropriate use of antibiotics in treating COPD exacerbations.

## Supporting information

S1 File Methods. Additional information on methodsi
(DOCX)

S2 File Data. Additional data including baseline demographics, G and F qPCR results, Clinical data for all visits and episodes, culture and pathogen-directed qPCR results for all visits by G:F cluster, and Virus positive data for day 0.
(DOCX)

## Abbreviations

ANOVA: One way analysis of variance
COPD: Chronic Obstructive Pulmonary Disease
CRP: C reactive protein
FEV_1_: Forced Expiratory Volume in 1 second
GOLD: Global initiative for chronic Obstructive Lung Disease
G:F: Gammaproteobacteria to Firmicutes ratio
HF: High Firmicutes subgroup
HG: High Gammproteobacteria subgroup
OTU: operational taxonomic unit
PCoA: Principle co-ordinate analysis
PERMANOVA: Permutation multivariate analysis of variance
PCR: polymerase chain reaction
GF: Balanced Gammaproteobacteria Firmicutes subgroup
QIIME: Quantitative Insights Into Microbial Ecology
qPCR: quantitative polymerase chain reaction
SEM: Standard error mean
