# Supplementary material for: Microbiome balance in sputum determined by PCR stratifies COPD exacerbations and shows potential for selective use of antibiotics
Source: PLoS One. 2017 Aug 25;12(8):e0182833. doi: 10.1371/journal.pone.0182833 (PMC5571965; doi:10.1371/journal.pone.0182833)
Supplement: S1 File — Additional information on methodsi (DOCX) [file pone.0182833.s001.docx]

Contents

[**Supporting information (Methods)** 2](#_Toc490755699)

[**Formula A: Calculation of Euclidean Distance measure between two exacerbation episodes** 2](#_Toc490755700)

[**Table A: Insilico analysis of published primers targeting Firmicutes and Gammaproteobacteria for sensitivity and specificity** 3](#_Toc490755701)

[**Figure A: Performance of Firmicutes and ϒproteo primer pairs in qPCR of mixed cultures** 5](#_Toc490755702)

[**Figure B: Correlation of qPCR derived ratio with 454 sequencing derived ratio** 6](#_Toc490755703)

# **Supporting information (Methods)**

## **Formula A: Calculation of Euclidean Distance measure between two exacerbation episodes**

D_Ee1, Ee2_ =$\sqrt{\left( S_{1}-S_{2} \right)^{2}+\left( {Day0}_{1}-{Day0}_{2} \right)^{2}+\left( {Day14}_{1}-{SDay14}_{2} \right)^{2}+\left( {Day42}_{1}-{Day42}_{2} \right)^{2}}$

Where

Ee1, Ee2 = exacerbation episode no

S_1_, S_2_ = stable visit samples

Day0_1_, Day0_2_ = exacerbation visit samples

Day14_1_, Day14_2_ = Post exacerbation Day 14 samples

Day42_1_, Day42_2_ = Post exacerbation Day 42 sample

| **Primer** | **Target Phylum** | **Sensitivity** *[hits in the cart (%)]* | **Specificity**  *[% hits inside cart/ouside cart(RDP 16S rRNA gene)]* | **% GammaProteobacteria seq (RDP)** | **% Streptococcus sequences (RDP)** | **References** |
| --- | --- | --- | --- | --- | --- | --- |
| **Firm 928F** | Firmicutes *( tested for sensitivity and specificity against RDP-Firmicutes 440938 sequences)* | 64.78 | 41.23 | 5.3 [Moraxella 0.04%,Pseudomonas(0.05%)] | 63 | (1) |
| **Firm 1040R** |  | 57.91 | 72.46 | 0.08 | 47 |  |
| Firm 934F |  | 55.03 | 54.63 | 14 [Pseudomonas 65%] | 53 | (2) |
| Firm 1060R |  | 61.67 | 58.69 | 0.09 | 47 |  |
| Firm 1063F |  | 65.9 | 27.7 | 40 [Haemophillus 1.5%,Moraxella 80%,Pseudomonas 67%] | 47 | (3) |
| Firm 1224R |  | 64.25 | 33.28 | 58 [Haemophilus 54%,Moraxella 86%,Pseudomonas65%] | 46 |  |
| Firm probe |  | 63.88 | 48.5 | 0.18 | 46 |  |
|  |  |  |  | **% Firmicutes seq(RDP)** | **% Haemophilus,Moraxella, Pseudomonas** |  |
| **ϒproteo 871F** | Gammaproteobactreia *(tested for sensitivity and specificity against RDP-Firmicutes 215820 sequences)* | 42.35 | 80.88 | 0.01 | Haemophilus(90), Moraxella(93),Pseudomonas(57) | (4) |
| ϒproteo 1080F |  | 60.5 | 11.77 | 66 [Streptococcus 46%] | Haemophilus(55),Moraxella(86), Pseudomonas(67) | (1) |
| ϒproteo 946F |  | 46.38 | 77.12 | 0.04 | Haemophilus(67)Moraxella(89)Pseudomonas(55) | (5) |
| **ϒproteo 1202R** |  | 51.32 | 54.24 | 0.04 | Haemophilus(54),Moraxella(86),Pseudomonas(65) | (1) |

## **Table A: Insilico analysis of published primers targeting Firmicutes and Gammaproteobacteria for sensitivity and specificity**

References

1. Bacchetti De Gregoris T, Aldred N, Clare AS, Burgess JG. Improvement of phylum- and class-specific primers for real-time PCR quantification of bacterial taxa. *J Microbiol Methods* 2011; 86: 351-356.

2. Guo X, Xia X, Tang R, Zhou J, Zhao H, Wang K. Development of a real-time PCR method for Firmicutes and Bacteroidetes in faeces and its application to quantify intestinal population of obese and lean pigs. *Lett Appl Microbiol* 2008; 47: 367-373.

3. Armougom F, Raoult D. Use of pyrosequencing and DNA barcodes to monitor variations in Firmicutes and Bacteroidetes communities in the gut microbiota of obese humans. *BMC Genomics* 2008; 9: 576.

4. Mühling M, Woolven-Allen J, Murrell JC, Joint I. Improved group-specific PCR primers for denaturing gradient gel electrophoresis analysis of the genetic diversity of complex microbial communities. *ISME J* 2008; 2: 379-392.

5. Klein AN, Frigon D, Raskin L. Populations related to Alkanindiges, a novel genus containing obligate alkane degraders, are implicated in biological foaming in activated sludge systems. *Environ Microbiol* 2007; 9: 1898-1912.

## **Figure A: Performance of Firmicutes and ϒproteo primer pairs in qPCR of mixed cultures**

Pure culture DNA, standardized at 10^7^ copies/μl, of *S. pneumoniae* (SP) and *H. influenzae* (HI) were used for dilutions and control for both qPCR assay. For the Firmicutes assay (upper) SP was the target and HI the non-target and vice versa for the ϒproteo assay (below). In both assays the target bacterium was diluted in equal proportion and in 10 fold dilution series up to 10^-4^ with the neat non-target bacterial DNA. Diluted target DNA was also used as control in the assays. X axis shows the name of the culture and dilution. The bars in red shows the calculated readings of target DNA in undiluted and in dilution mixes with non-target DNA and, undiluted non-target DNA Blue bars represent the observed readings of the target and non-target DNA in these assays.

## **Figure B: Correlation of qPCR derived ratio with 454 sequencing derived ratio**

Dotted lines represent the 95%CI.
