# Supplementary material for: Microbiome balance in sputum determined by PCR stratifies COPD exacerbations and shows potential for selective use of antibiotics
Source: PLoS One. 2017 Aug 25;12(8):e0182833. doi: 10.1371/journal.pone.0182833 (PMC5571965; doi:10.1371/journal.pone.0182833)
Supplement: S2 File — Additional data including baseline demographics, G and F qPCR results, Clinical data for all visits and episodes, culture and pathogen-directed qPCR results for all visits by G:F cluster, and Virus positive data for day 0. (DOCX) [file pone.0182833.s002.docx]

**Supporting Information – (Data)**

**NB – All large tables are directly compatible with spreadsheets for easier viewing.**

Contents

[**Supporting Information – (Data)** 1](#_Toc490755817)

[**Figure A: Increases in the G:F ratio at exacerbation (day 0) in the HG group reflect contemporaneous shifts in both G and F components.** 2](#_Toc490755818)

[**Table A: Demographics and baseline data of 58 subjects** 3](#_Toc490755819)

[**Table B: Quantification data from Gammaproteobacteria and Firmicutes qPCR** 9](#_Toc490755820)

[**Table C: Clinical data across the 4 visit times in 66 exacerbation episodes** 16](#_Toc490755821)

[**Table D: Culture and qPCR readings across the 4 visit samples in the G:F cluster** 20](#_Toc490755822)

[**Table E: Virus positive exacerbation (day 0) visits** 26](#_Toc490755823)

**Figure A: Increases in the G:F ratio at exacerbation (day 0) in the HG group reflect contemporaneous shifts in both G and F components.**

Separated results for Gammaproteobateria and Firmicute analyses presented for the HG group. Significance values represent paired t-tests.

**Table A: Demographics and baseline data of 58 subjects**

| **Exacerbation episode No** | **P:Fcluster** | **Sex** | **Smoking_status** | **PYH** | **Age** | **H** | **W** | **BMI** | **E_Freq** | **Blood CRP** | **Sputum Neutrophil (%)** | **FVC** | **FEV1** | **FEV1/FVC %** | **FEV_postBD** | **predicted FEV** | **PostBD**  **%pred** | **GOLD** | **SGRQ_Total** | **BDP** |
| --- | --- | --- | --- | --- | --- | --- | --- | --- | --- | --- | --- | --- | --- | --- | --- | --- | --- | --- | --- | --- |
| 1 | HF | Male | Ex-smoker | 37.5 | 86.89 | 184 | 103 | 30.42 | 3 | 3 | 12.93 | 1.9 | 1.5 | 0.79 | 1.5 | 2.87 | 52.26 | 2 | 71.78 | 2000 |
| 2 | PF | Male | Ex-smoker | 48 | 69.10 | 177 | 84 | 26.81 | 3 | 3 | 60.5 | 2.7 | 0.8 | 0.30 | 0.95 | 3.09 | 30.74 | 3 | 43.58 | 1000 |
| 3 | PF | Female | Ex-smoker | 51 | 76.05 | 162 | 88 | 33.53 | 1 |  | 67.32 | 2.1 | 1.35 | 0.64 | 1.45 | 1.87 | 77.54 | 2 | 71.39 | 2000 |
| 4 | HF | Male | Habitual smoker | 31.5 | 62.09 | 173 | 77 | 25.73 | 3 | 10 | 37 | 3.75 | 1.65 | 0.44 | 1.9 | 3.12 | 60.9 | 2 | 86.53 | 2000 |
| 5 | HF | Male | Ex-smoker | 22.5 | 84.12 | 170 | 68 | 23.53 | 4 | 3 | 84.5 | 2 | 0.95 | 0.48 | 0.98 | 2.36 | 41.53 | 3 |  | 800 |
| 6 | PF | Male | Habitual smoker | 49 | 64.74 | 170 | 65 | 22.49 | 3 | 19 | 90.25 | 2 | 1.05 | 0.53 | 1 | 2.94 | 34.01 | 3 | 59.21 | 2000 |
| 7 | HF | Female | Habitual smoker | 34 | 46.55 | 162 | 52 | 19.81 | 9 | 3 | 71.75 | 4.25 | 2.65 | 0.62 | 2.4 | 2.87 | 83.62 | 1 | 33.36 | 400 |
| 8 | HG | Male | Ex-smoker | 51.3 | 83.40 | 177 | 78 | 24.90 | 5 | 3 | 85.5 | 2.8 | 1.81 | 0.65 | 1.78 | 2.69 | 66.17 | 2 | 15.4 |  |
| 9 | HG | Male | Ex-smoker | 63.75 | 76.51 | 160 | 82 | 32.03 | 4 | 3 |  | 2.3 | 1.25 | 0.54 | 1.4 | 2.16 | 64.81 | 2 | 64.16 | 2000 |
| 10 | PF | Male | Habitual smoker | 60 | 71.38 | 168 | 58 | 20.55 | 1 | 3 | 85.5 | 2.21 | 0.6 | 0.27 | 0.6 | 2.65 | 22.64 | 4 | 32.25 | 1000 |
| 11 | HF | Male | Habitual smoker | 65 | 78.54 | 176 | 72 | 23.24 | 2 | 5 | 68 | 2.56 | 1.26 | 0.49 | 1.29 | 2.79 | 46.24 | 3 | 43.49 | 2000 |
| 12 | HF | Female | Ex-smoker | 9.5 | 72.44 | 168 | 65 | 23.03 | 7 | 5 | 37.58 | 2.2 | 1.45 | 0.66 | 1.42 | 2.21 | 64.25 | 2 | 36.49 | 1600 |
| 13 | HF | Male | Ex-smoker | 42 | 65.74 | 180 | 95 | 29.32 | 8 | 9 | 55.02 | 3.2 | 1.15 | 0.36 | 1.25 | 3.34 | 37.43 | 3 | 64.46 | 2000 |
| 14 | HF | Male | Ex-smoker | 22 | 77.61 | 167 | 57 | 20.44 | 10 | 6 | 34.02 | 2.05 | 0.65 | 0.32 | 0.85 | 2.43 | 34.98 | 3 | 49.38 | 2000 |
| 15 | HG | Male | Ex-smoker | 6 | 64.64 | 162 | 94 | 35.82 | 0 | 14 | 82.25 | 3.26 | 1.7 | 0.52 | 2.25 | 2.95 | 76.27 | 2 | 31.11 |  |
| 16 | HF | Male | Ex-smoker | 13.5 | 72.36 | 164 | 70 | 26.03 | 3 | 3 | 71 | 2.9 | 1.55 | 0.53 | 1.7 | 2.45 | 69.39 | 2 | 55.15 | 1600 |
| 17 | HF | Male | Habitual smoker | 60 | 60.94 | 171 | 64 | 21.89 | 6 | 3 | 96.5 | 1.16 | 0.54 | 0.47 | 0.66 | 3.09 | 21.36 | 4 | 59.22 | 1000 |
| 18 | HF | Male | Ex-smoker | 120 | 65.25 | 168 | 85 | 30.12 | 3 | 7 | 32.43 | 2.58 | 1.6 | 0.62 | 1.66 | 2.82 | 58.87 | 2 | 74.81 | 800 |
| 19 | PF | Male | Habitual smoker | 106 | 67.70 | 168 | 72 | 25.51 | 1 |  | 81.75 | 3 | 2.05 | 0.68 | 2.2 | 2.76 | 79.71 | 2 | 36.9 | 1000 |
| 20 | HG | Male | Ex-smoker | 17.5 | 75.40 | 176 | 68 | 21.95 | 6 | 3 | 95.75 | 3 | 1 | 0.33 | 1.1 | 3.02 | 36.42 | 3 |  | 2000 |
| 21 | HG | Male | Habitual smoker | 42 | 71.02 | 181 | 92 | 28.08 | 2 | 7 | 35.5 | 4.2 | 2.2 | 0.52 | 2.1 | 3.23 | 65.02 | 2 | 42.07 | 1000 |
| 22 | HF | Male | Ex-smoker | 18.75 | 79.94 | 168 | 64 | 22.68 | 9 | 3 | 45.25 | 1.9 | 1 | 0.53 | 1.15 | 2.41 | 47.72 | 3 | 67.61 | 2000 |
| 23 | HF | Female | Ex-smoker | 32.25 | 72.94 | 155 | 70 | 29.14 | 1 | 30 | 72.75 | 1.89 | 0.54 | 0.29 | 0.61 | 1.7 | 35.88 | 3 | 53.15 | 2000 |
| 24 | HG | Male | Ex-smoker | 106 | 64.75 | 181 | 74 | 22.59 | 2 | 3 | 84 | 3.72 | 1.42 | 0.38 | 1.68 | 3.41 | 49.27 | 3 | 27.04 | 2000 |
| 25 | HF | Male | Non-smoker | 0 | 66.54 | 178 | 121 | 38.19 | 6 | 3 | 94.25 | 2.3 | 0.7 | 0.30 | 0.6 | 3.22 | 18.63 | 4 | 50.85 | 2000 |
| 26 | PF | Male | Non-smoker | 0 | 62.00 | 180 | 82 | 25.31 | 0 | 13 | 94.5 | 4.18 | 2.32 | 0.56 | 2.37 | 3.45 | 68.7 | 2 | 16.64 | 2000 |
| 27 | HF | Female | Ex-smoker | 21 | 66.91 | 156 | 49 | 20.13 | 3 | 8 | 90.25 | 1.75 | 0.75 | 0.43 | 0.67 | 1.89 | 35.45 | 3 | 39.7 | 2000 |
| 28 | HF | Female | Ex-smoker | 36.9 | 64.25 | 161.5 | 67 | 25.69 | 7 | 3 | 67.89 | 1.65 | 0.83 | 0.50 | 0.9 | 2.2 | 40.91 | 3 | 62.61 | 800 |
| 29 | HF | Male | Habitual smoker | 45 | 53.53 | 172.5 | 68 | 22.85 | 4 | 3 | 89.5 | 3.6 | 1.9 | 0.53 | 1.95 | 3.38 | 57.69 | 2 | 44.35 | 400 |
| 30 | PF | Male | Ex-smoker | 60 | 67.60 | 186 | 74 | 21.39 | 4 | 8 | 68.97 | 3.2 | 0.95 | 0.30 | 0.95 | 3.54 | 26.84 | 4 | 30.83 | 2000 |
| 06b | HG | Female | Habitual smoker | 46 | 61.17 | 164 | 68 | 25.28 | 1 | 3 | 69.25 | 2.34 | 0.89 | 0.38 | 1.14 | 2.33 | 48.93 | 3 | 30.34 | 1000 |
| 23b | HG | Male | Ex-smoker | 26 | 70.71 | 180 | 90 | 27.78 | 2 | 3 | 53.25 |  |  |  |  | 3.19 |  |  | 56.01 | 400 |
| 26b | HF | Male | Ex-smoker | 38 | 60.02 | 171 | 98 | 33.51 | 5 | 3 | 78.96 | 2.6 | 1.55 | 0.60 | 1.95 | 3.12 | 62.5 | 2 | 66.99 | 2000 |
| 03_ph2 | HF | male | Ex- smoker | 33 | 76.57 | 166 | 90 | 32.66 | 3 | 18 | 94.5 | 1.34 | 0.52 | 0.39 | 0.51 | 2.44 | 20.9 | 4 | 82.85 | 2000 |
| 04_ph2 | out | Male | Ex Smoker | 51 | 62.95 | 180 | 67 | 20.68 | 6 | 78 | 89.5 | 3.19 | 0.96 | 0.30 | 1.12 | 3.43 | 32.65 | 3 | 42.86 | 2000 |
| 07_ph2 | HG | Female | Ex- Smoker | 48 | 62.92 | 164 | 73 | 27.14 | 2 | 3 | 97 | 3.19 | 1.29 | 0.40 | 1.31 | 2.33 | 56.22 | 2 | 34.47 | 2000 |
| 08_ph2 | HF | Male | Ex- Smoker | 11.5 | 78.65 | 177 | 95 | 30.32 | 2 | 3 | 68.25 | 3.4 | 1.73 | 0.51 | 1.78 | 2.86 | 62.24 | 2 | 45.31 | 800 |
| 10_ph2 | HF | Male | Habitual smoker | 99 | 80.24 | 174 | 72 | 23.78 | 1 | 19 | 88 | 2.43 | 1.19 | 0.49 | 1.11 | 2.79 | 39.78 | 3 | 44.39 | 800 |
| 11_ph2 | HF | Male | Habitual smoker | 61 | 71.03 | 178 | 74 | 23.36 | 3 | 6 | 88 | 2.46 | 0.91 | 0.37 |  | 3.11 |  |  | 79.24 | 2000 |
| 12_ph2 | HG | Female | Ex- Smoker | 36 | 78.84 | 149 | 66 | 29.73 | 1 | 3 | 82.75 | 1.45 | 0.65 | 0.45 | 0.61 | 1.34 | 45.52 | 3 | 45.24 | 1000 |
| 13_ph2 | HF | male | Ex- Smoker | 55 | 79.13 | 167 | 59 | 21.16 | 3 | 3 | 40 | 1.45 | 0.6 | 0.41 | 0.6 | 2.39 | 25.1 | 4 | 44.07 | 2000 |
| 14_ph2 | HF | Male | Ex- Smoker | 92 | 66.84 | 168 | 85 | 30.12 | 1 | 3 | 69.73 | 2.41 | 1.52 | 0.63 | 1.56 | 2.82 | 55.32 | 2 | 49.41 | 400 |
| 15_ph2 | out | Female | Habitual smoker | 49 | 66.02 | 165 | 70 | 25.71 | 3 | 3 | 20 | 2.48 | 1.12 | 0.45 |  | 2.27 |  |  | 72.03 | 400 |
| 21_ph2 | HG | Male | Non-Smoker | 0 | 63.47 | 180 | 76 | 23.46 | 1 | 3 | 99 | 3.96 | 2.3 | 0.58 | 2.46 | 3.42 | 71.93 | 2 | 19.93 | 1000 |
| 22_ph2 | HG | male | Ex- Smoker | 78 | 78.60 | 172 | 83 | 28.06 | 1 | 14 | 68.75 | 3.9 | 2.33 | 0.60 | 2.4 | 2.62 | 91.6 | 1 | 26.43 |  |
| 24_ph2 | PF | Male | Ex- Smoker | 35 | 66.13 | 165 | 88 | 32.32 | 1 | 15 | 94.25 | 2.9 | 1.12 | 0.39 | 1.23 | 2.69 | 45.72 | 3 | 46.86 | 800 |
| 25_ph2 | PF | Male | Ex- Smoker | 10 | 65.70 | 166 | 71 | 25.77 | 4 | 3 | 98.75 | 3.67 | 1.73 | 0.47 | 1.76 | 2.74 | 64.23 | 2 | 56.04 | 2000 |
| 27_ph2 | HG | male | Habitual smoker | 50 | 81.19 | 178 | 70 | 22.09 | 1 | 3 | 26.75 | 3.31 | 2.08 | 0.63 | 1.91 | 2.82 | 67.73 | 2 | 51.42 | 1000 |
| 28_ph2 | HG | male | Habitual smoker | 80 | 50.58 | 175 | 108 | 35.27 | 1 | 10 | 88.25 | 2.32 | 0.85 | 0.37 | 1.03 | 3.59 | 28.69 | 4 | 77.44 | 1000 |
| 29_ph2 | HF | Male | Habitual smoker | 41 | 62.18 | 155 | 50 | 20.81 | 1 | 3 | 91.75 | 2.47 | 1.19 | 0.48 | 1.28 | 2.38 | 53.78 | 2 | 50 |  |
| 30_ph2 | HG | male | Ex- smoker | 37 | 46.63 | 173 | 76 | 25.39 | 4 | 3 | 96.75 | 2.63 | 0.94 | 0.36 | 0.93 | 3.7 | 25.14 | 4 | 53.9 | 400 |
| 31_ph2 | HF | Male | Habitual smoker | 49 | 63.86 | 165 | 75 | 27.55 | 2 | 5 | 36.5 | 3.14 | 1.29 | 0.41 | 1.44 | 2.76 | 52.17 | 2 |  |  |
| 32_ph2 | HG | Female | Habitual smoker | 33 | 61.06 | 161 | 62 | 23.92 | 1 | 3 | 47 | 2.11 | 1.11 | 0.53 | 1.3 | 2.39 | 54.39 | 2 | 53.48 | 800 |
| 34_ph2 | HF | Male | Habitual smoker | 61 | 77.21 | 175 | 75 | 24.49 | 1 | 9 | 32.75 | 3.9 | 1.8 | 0.46 | 1.6 | 2.78 | 57.55 | 2 | 53.07 |  |
| 35_ph2 | HF | Male | Habitual smoker | 41 | 56.53 | 173 | 102 | 34.08 | 1 | 7 | 59.5 | 2.23 | 0.7 | 0.31 | 0.76 | 3.33 | 22.82 | 4 | 62.67 | 800 |
| 36_ph2 | HF | Female | Habitual smoker | 49 | 66.73 | 160 | 97 | 37.89 | 5 | 33 | 95 | 1.47 | 0.65 | 0.44 | 0.65 | 2.05 | 31.71 | 3 | 66.69 | 2000 |
| 37_ph2 | HF | Male | Habitual smoker | 57 | 75.52 | 175 | 82 | 26.78 | 4 | 27 | 41.5 | 3.35 | 2.05 | 0.61 | 2.1 | 2.84 | 73.94 | 2 | 59.99 | 2000 |
| 38_ph2 | HG | Male | Habitual smoker | 56 | 63.87 | 172 | 76 | 25.69 | 3 | 3 | 70.75 | 3.58 | 1.39 | 0.39 | 1.64 | 3.08 | 53.25 | 2 | 95.09 | 2000 |

H = height; W= weight; BMI= Body mass index; PHY = Pack year history; E_Freq = exacerbation frequency in past 12 months; CRP=C reactive protein; FEV_1_=forced expiratory volume in 1 second; FVC=forced vital capacity; FEV_1_, % predicted = Spirometry recorded post bronchodilator; SGRQ= St George’s Respiratory Questionnaire; BDP=beclometasone dipropionate ( inhaled corticosteroid).

**Table B: Quantification data from Gammaproteobacteria and Firmicutes qPCR**

| **Exacerbation episode no** | **Phase_Subject_VisitID** | **Sample no** | **G(copies/ul)** | **F(copies/ul)** | **Ratio** | **Log(Ratio)** |
| --- | --- | --- | --- | --- | --- | --- |
| 1 | 1_2_3 | S1 | 7.31E+03 | 1.53E+04 | 0.48 | -1.06 |
|  | 1_2_3.1 | E1 | 5.29E+04 | 5.77E+04 | 0.92 | -0.13 |
|  | 1_2_3.11 | F1 | 1.12E+05 | 1.12E+06 | 0.10 | -3.33 |
|  | 1_2_3.12 | R1 | 8.46E+03 | 8.25E+03 | 1.03 | 0.04 |
| 2 | 1_3_2 | S2 | 6.08E+05 | 6.03E+05 | 1.01 | 0.01 |
|  | 1_3_2.1 | E2 | 3.36E+05 | 1.82E+05 | 1.85 | 0.89 |
|  | 1_3_2.11 | F2 | 2.96E+04 | 8.68E+03 | 3.41 | 1.77 |
|  | 1_3_2.12 | R2 | 3.47E+06 | 2.84E+02 | 12227.04 | 13.58 |
| 3 | 1_7_4 | S3 | 4.72E+06 | 8.17E+06 | 0.58 | -0.79 |
|  | 1_7_4.1 | E3 | 2.13E+07 | 2.38E+06 | 8.96 | 3.16 |
|  | 1_7_4.11 | F3 | 8.83E+07 | 2.99E+04 | 2955.67 | 11.53 |
|  | 1_7_4.12 | R3 | 9.61E+07 | 1.07E+05 | 895.91 | 9.81 |
| 4 | 1_9_5 | S4 | 9.49E+05 | 1.97E+04 | 48.28 | 5.59 |
|  | 1_9_5.1 | E4 | 3.26E+05 | 6.85E+04 | 4.75 | 2.25 |
|  | 1_9_5.11 | F4 | 4.73E+05 | 3.60E+05 | 1.31 | 0.39 |
|  | 1_9_5.12 | R4 | 6.13E+04 | 1.52E+05 | 0.40 | -1.31 |
| 5 | 1_21_5 | S5 | 1.19E+05 | 3.01E+05 | 0.40 | -1.34 |
|  | 1_21_5.1 | E5 | 6.18E+05 | 6.08E+06 | 0.10 | -3.30 |
|  | 1_21_5.11 | F5 | 5.28E+05 | 8.19E+05 | 0.64 | -0.63 |
|  | 1_21_5.12 | R5 | 1.19E+06 | 2.71E+05 | 4.39 | 2.13 |
| 6 | 1_23_2 | S6 | 1.40E+09 | 2.69E+05 | 5227.02 | 12.35 |
|  | 1_23_2.1 | E6 | 5.45E+07 | 6.59E+03 | 8267.02 | 13.01 |
|  | 1_23_2.11 | F6 | 2.76E+08 | 1.35E+04 | 20525.06 | 14.33 |
|  | 1_23_2.12 | R6 | 1.44E+09 | 2.00E+07 | 72.26 | 6.18 |
| 7 | 1_27_3 | S7 | 2.93E+05 | 6.12E+05 | 0.48 | -1.06 |
|  | 1_27_3.1 | E7 | 6.65E+05 | 5.38E+06 | 0.12 | -3.02 |
|  | 1_27_3.11 | F7 | 9.81E+04 | 5.47E+04 | 1.80 | 0.84 |
|  | 1_27_3.12 | R7 | 5.12E+05 | 9.24E+05 | 0.55 | -0.85 |
| 8 | 1_76_4 | S8 | 4.52E+08 | 3.15E+06 | 143.68 | 7.17 |
|  | 1_76_4.2 | E8 | 1.91E+07 | 4.73E+04 | 404.24 | 8.66 |
|  | 1_76_4.21 | F8 | 1.40E+05 | 5.07E+05 | 0.28 | -1.86 |
|  | 1_76_4.22 | R8 | 9.27E+07 | 1.05E+07 | 8.79 | 3.14 |
| 9 | 1_39_2 | S9 | 2.60E+06 | 2.22E+06 | 1.17 | 0.23 |
|  | 1_39_2.2 | E9 | 1.35E+08 | 8.34E+04 | 1616.48 | 10.66 |
|  | 1_39_2.21 | F9 | 3.64E+05 | 1.59E+05 | 2.30 | 1.20 |
|  | 1_39_2.22 | R9 | 3.08E+05 | 2.91E+04 | 10.58 | 3.40 |
| 10 | 1_40_5 | S10 | 4.44E+06 | 4.44E+05 | 10.01 | 3.32 |
|  | 1_40_5.1 | E10 | 8.49E+06 | 9.09E+05 | 9.33 | 3.22 |
|  | 1_40_5.11 | F10 | 9.88E+06 | 3.38E+04 | 292.83 | 8.19 |
|  | 1_40_5.12 | R10 | 1.60E+07 | 7.76E+04 | 206.11 | 7.69 |
| 11 | 1_44_5 | S11 | 5.40E+05 | 8.52E+05 | 0.63 | -0.66 |
|  | 1_44_5.1 | E11 | 2.53E+04 | 1.52E+05 | 0.17 | -2.59 |
|  | 1_44_5.11 | F11 | 2.57E+04 | 1.29E+06 | 0.02 | -5.65 |
|  | 1_44_5.12 | R11 | 6.91E+04 | 4.97E+05 | 0.14 | -2.84 |
| 12 | 1_47_2 | S12 | 8.34E+03 | 5.82E+04 | 0.14 | -2.80 |
|  | 1_47_2.1 | E12 | 1.32E+05 | 9.42E+04 | 1.40 | 0.49 |
|  | 1_47_2.11 | F12 | 1.41E+04 | 5.13E+04 | 0.28 | -1.86 |
|  | 1_47_2.12 | R12 | 2.30E+05 | 2.28E+06 | 0.10 | -3.31 |
| 13 | 1_52_2 | S13 | 4.25E+06 | 2.24E+03 | 1900.05 | 10.89 |
|  | 1_52_2.2 | E13 | 1.97E+05 | 3.92E+05 | 0.50 | -0.99 |
|  | 1_52_2.21 | F13 | 1.95E+06 | 3.77E+05 | 5.16 | 2.37 |
|  | 1_52_2.22 | R13 | 1.03E+05 | 6.23E+04 | 1.66 | 0.73 |
| 14 | 1_56_2 | S14 | 1.66E+04 | 7.92E+03 | 2.10 | 1.07 |
|  | 1_56_2.1 | E14 | 1.81E+05 | 2.27E+05 | 0.80 | -0.33 |
|  | 1_56_2.11 | F14 | 2.70E+05 | 1.06E+05 | 2.55 | 1.35 |
|  | 1_56_2.12 | R14 | 1.20E+05 | 1.34E+05 | 0.90 | -0.15 |
| 15 | 1_57_2 | S15 | 2.15E+06 | 8.63E+05 | 2.49 | 1.32 |
|  | 1_57_2.1 | E15 | 2.45E+06 | 4.01E+04 | 61.11 | 5.93 |
|  | 1_57_2.11 | F15 | 6.89E+05 | 2.79E+05 | 2.47 | 1.31 |
|  | 1_57_2.12 | R15 | 1.99E+05 | 3.40E+04 | 5.86 | 2.55 |
| 16 | 1_58_2 | S16 | 2.41E+05 | 1.90E+06 | 0.13 | -2.98 |
|  | 1_58_2.1 | E16 | 2.96E+04 | 2.22E+05 | 0.13 | -2.91 |
|  | 1_58_2.11 | F16 | 7.92E+05 | 5.09E+03 | 155.61 | 7.28 |
|  | 1_58_2.12 | R16 | 3.26E+05 | 6.90E+04 | 4.72 | 2.24 |
| 17 | 1_60_2 | S17 | 6.28E+06 | 1.12E+05 | 56.29 | 5.81 |
|  | 1_60_2.1 | E17 | 1.35E+06 | 3.62E+05 | 3.75 | 1.91 |
|  | 1_60_2.11 | F17 | 2.70E+04 | 8.09E+03 | 3.34 | 1.74 |
|  | 1_60_2.12 | R17 | 1.45E+06 | 7.28E+04 | 19.95 | 4.32 |
| 18 | 1_61_3 | S18 | 3.47E+06 | 9.99E+06 | 0.35 | -1.53 |
|  | 1_61_3.1 | E18 | 2.96E+07 | 2.36E+07 | 1.25 | 0.33 |
|  | 1_61_3.11 | F18 | 1.46E+06 | 5.56E+05 | 2.62 | 1.39 |
|  | 1_61_3.12 | R18 | 8.28E+04 | 7.46E+04 | 1.11 | 0.15 |
| 19 | 1_63_4 | S19 | 5.39E+07 | 6.19E+05 | 87.19 | 6.45 |
|  | 1_63_4.1 | E19 | 1.05E+07 | 6.55E+04 | 161.03 | 7.33 |
|  | 1_63_4.11 | F19 | 6.72E+07 | 2.39E+05 | 281.45 | 8.14 |
|  | 1_63_4.12 | R19 | 2.16E+07 | 8.27E+04 | 261.59 | 8.03 |
| 20 | 1_71_2 | S20 | 1.52E+04 | 2.89E+02 | 52.46 | 5.71 |
|  | 1_71_2.1 | E20 | 2.63E+05 | 5.55E+02 | 473.52 | 8.89 |
|  | 1_71_2.11 | F20 | 7.28E+05 | 7.79E+04 | 9.35 | 3.23 |
|  | 1_71_2.12 | R20 | 2.51E+04 | 1.87E+04 | 1.34 | 0.42 |
| 22 | 1_78_2 | S22 | 3.07E+06 | 2.03E+06 | 1.51 | 0.60 |
|  | 1_78_2.1 | E22 | 4.41E+05 | 4.16E+06 | 0.11 | -3.24 |
|  | 1_78_2.11 | F22 | 5.41E+06 | 2.47E+06 | 2.19 | 1.13 |
|  | 1_78_2.12 | R22 | 1.06E+07 | 2.89E+06 | 3.67 | 1.88 |
| 24 | 1_83_2 | S24 | 8.83E+06 | 2.41E+06 | 3.66 | 1.87 |
|  | 1_83_2.1 | E24 | 9.44E+06 | 5.25E+03 | 1796.54 | 10.81 |
|  | 1_83_2.11 | F24 | 1.50E+05 | 1.02E+04 | 14.71 | 3.88 |
|  | 1_83_2.12 | R24 | 1.75E+06 | 1.51E+06 | 1.15 | 0.21 |
| 25 | 1_93_2 | S25 | 1.28E+05 | 9.61E+04 | 1.33 | 0.41 |
|  | 1_93_2.1 | E25 | 2.03E+05 | 3.74E+04 | 5.42 | 2.44 |
|  | 1_93_2.11 | F25 | 8.05E+04 | 1.55E+05 | 0.52 | -0.95 |
|  | 1_93_2.12 | R25 | 4.44E+03 | 7.73E+03 | 0.57 | -0.80 |
| 26 | 1_96_4 | S26 | 3.33E+08 | 2.62E+05 | 1271.17 | 10.31 |
|  | 1_96_4.1 | E26 | 2.36E+07 | 2.55E+05 | 92.55 | 6.53 |
|  | 1_96_4.11 | F26 | 1.20E+07 | 1.06E+04 | 1139.88 | 10.15 |
|  | 1_96_4.12 | R26 | 1.45E+07 | 1.90E+04 | 763.91 | 9.58 |
| 27 | 1_102_3 | S27 | 6.16E+03 | 1.13E+04 | 0.55 | -0.87 |
|  | 1_102_3.1 | E27 | 1.83E+05 | 4.47E+04 | 4.10 | 2.04 |
|  | 1_102_3.11 | F27 | 2.37E+04 | 9.49E+03 | 2.50 | 1.32 |
|  | 1_102_3.12 | R27 | 3.28E+05 | 2.95E+05 | 1.11 | 0.15 |
| 28 | 1_115_2 | S28 | 1.91E+07 | 1.54E+06 | 12.40 | 3.63 |
|  | 1_115_2.1 | E28 | 1.10E+05 | 6.44E+05 | 0.17 | -2.55 |
|  | 1_115_2.11 | F28 | 7.95E+06 | 4.77E+07 | 0.17 | -2.59 |
|  | 1_115_2.12 | R28 | 3.43E+05 | 1.59E+06 | 0.22 | -2.21 |
| 29 | 1_122_3 | S29 | 5.01E+07 | 3.29E+07 | 1.52 | 0.61 |
|  | 1_122_3.2 | E29 | 9.22E+07 | 2.08E+07 | 4.43 | 2.15 |
|  | 1_122_3.21 | F29 | 1.13E+08 | 1.00E+07 | 11.30 | 3.50 |
|  | 1_122_3.22 | R29 | 2.57E+05 | 8.03E+05 | 0.32 | -1.65 |
| 30 | 1_123_2 | S30 | 6.97E+07 | 1.20E+06 | 57.99 | 5.86 |
|  | 1_123_2.1 | E30 | 5.03E+07 | 1.66E+06 | 30.28 | 4.92 |
|  | 1_123_2.11 | F30 | 2.47E+08 | 1.21E+07 | 20.43 | 4.35 |
|  | 1_123_2.12 | R30 | 5.96E+09 | 3.75E+05 | 15892.94 | 13.96 |
| 01b | 1_2_4 | 1A | 1.65E+05 | 8.57E+06 | 0.02 | -5.70 |
|  | 1_2_4.1 | 1B | 1.25E+04 | 5.25E+04 | 0.24 | -2.07 |
|  | 1_2_4.11 | 1C | 1.11E+06 | 1.85E+06 | 0.60 | -0.73 |
|  | 1_2_4.12 | 1D | 3.19E+04 | 1.71E+05 | 0.19 | -2.42 |
| 02b | 1_2_5 | 2A | 5.25E+03 | 1.28E+04 | 0.41 | -1.29 |
|  | 1_2_5.1 | 2B | 7.98E+06 | 7.43E+04 | 107.35 | 6.75 |
|  | 1_2_5.11 | 2C | 3.76E+04 | 2.99E+04 | 1.26 | 0.33 |
|  | 1_2_5.12 | 2D | 1.24E+08 | 6.55E+05 | 189.98 | 7.57 |
| 05b | 1_27_4 | 5A | 5.26E+04 | 1.00E+05 | 0.52 | -0.93 |
|  | 1_27_4.1 | 5B | 5.30E+05 | 1.93E+06 | 0.27 | -1.87 |
|  | 1_27_4.11 | 5C | 1.23E+04 | 5.04E+05 | 0.02 | -5.35 |
|  | 1_27_4.12 | 5D | 8.46E+03 | 1.58E+05 | 0.05 | -4.22 |
| 06b | 1_31_4 | 6A | 6.58E+05 | 6.02E+05 | 1.09 | 0.13 |
|  | 1_31_4.1 | 6B | 8.19E+06 | 1.11E+05 | 73.98 | 6.21 |
|  | 1_31_4.11 | 6C | 3.86E+07 | 4.76E+06 | 8.11 | 3.02 |
|  | 1_31_4.12 | 6D | 1.44E+05 | 6.86E+04 | 2.09 | 1.07 |
| 09b | 1_39_5 | 9A | 1.70E+06 | 5.06E+05 | 3.36 | 1.75 |
|  | 1_39_5.1 | 9B | 2.75E+07 | 4.20E+03 | 6549.37 | 12.68 |
|  | 1_39_5.11 | 9C | 1.57E+05 | 4.94E+04 | 3.18 | 1.67 |
|  | 1_39_5.12 | 9D | 2.90E+04 | 1.48E+04 | 1.97 | 0.97 |
| 21 | 1_74_3 | 16A | 4.34E+05 | 3.23E+05 | 1.34 | 0.43 |
|  | 1_74_3.1 | 16B | 2.64E+07 | 6.82E+05 | 38.71 | 5.27 |
|  | 1_74_3.11 | 16C | 1.46E+05 | 8.92E+03 | 16.30 | 4.03 |
|  | 1_74_3.12 | 16D | 1.98E+05 | 4.29E+05 | 0.46 | -1.11 |
| 17b | 1_74_4 | 17A | 4.85E+04 | 4.10E+04 | 1.18 | 0.24 |
|  | 1_74_4.1 | 17B | 2.37E+03 | 3.24E+03 | 0.73 | -0.45 |
|  | 1_74_4.11 | 17C | 2.04E+05 | 9.20E+05 | 0.22 | -2.17 |
|  | 1_74_4.12 | 17D | 2.23E+06 | 4.85E+06 | 0.46 | -1.12 |
| 18b | 1_78_4 | 18A | 2.92E+05 | 2.49E+04 | 11.72 | 3.55 |
|  | 1_78_4.1 | 18B | 3.57E+05 | 2.41E+05 | 1.48 | 0.57 |
|  | 1_78_4.11 | 18C | 1.43E+06 | 3.25E+03 | 439.45 | 8.78 |
|  | 1_78_4.12 | 18D | 4.19E+06 | 5.01E+06 | 0.84 | -0.26 |
| 23 | 1_79_2 | 19A | 3.38E+05 | 3.83E+04 | 8.81 | 3.14 |
|  | 1_79_2.1 | 19B | 2.16E+05 | 2.06E+05 | 1.05 | 0.07 |
|  | 1_79_2.11 | 19C | 1.23E+06 | 8.06E+03 | 152.45 | 7.25 |
|  | 1_79_2.12 | 19D | 2.27E+06 | 3.26E+06 | 0.69 | -0.53 |
| 20b | 1_79_4 | 20A | 2.97E+05 | 1.39E+05 | 2.14 | 1.10 |
|  | 1_79_4.1 | 20B | 1.14E+05 | 1.53E+04 | 7.45 | 2.90 |
|  | 1_79_4.11 | 20C | 2.94E+05 | 4.64E+04 | 6.34 | 2.66 |
|  | 1_79_4.12 | 20D | 7.55E+04 | 9.82E+03 | 7.69 | 2.94 |
| 23b | 1_108_3 | 23A | 3.20E+05 | 1.56E+06 | 0.21 | -2.28 |
|  | 1_108_3.1 | 23B | 4.33E+05 | 1.12E+04 | 38.72 | 5.27 |
|  | 1_108_3.11 | 23C | 4.51E+03 | 5.89E+02 | 7.66 | 2.94 |
|  | 1_108_3.12 | 23D | 9.44E+03 | 7.13E+03 | 1.32 | 0.40 |
| 26b | 1_141_2 | 26A | 1.68E+05 | 2.55E+04 | 6.57 | 2.72 |
|  | 1_141_2.1 | 26B | 6.68E+05 | 3.44E+05 | 1.94 | 0.96 |
|  | 1_141_2.11 | 26C | 2.83E+06 | 6.34E+06 | 0.45 | -1.16 |
|  | 1_141_2.12 | 26D | 1.08E+06 | 2.60E+05 | 4.16 | 2.06 |
| 03_ph2 | 2_14_3 | 3A | 2.52E+06 | 1.55E+03 | 1629.36 | 10.67 |
|  | 2_14_3.1 | 3B | 6.01E+03 | 2.07E+05 | 0.03 | -5.11 |
|  | 2_14_3.11 | 3C | 2.66E+06 | 4.90E+05 | 5.42 | 2.44 |
|  | 2_14_3.12 | 3D | 2.38E+03 | 1.36E+04 | 0.17 | -2.52 |
| 04_ph2 | 2_19_2 | 4A | 1.75E+03 | 4.50E+05 | 0.00 | -8.01 |
|  | 2_19_2.1 | 4B | 6.55E+06 | 3.61E+02 | 18134.05 | 14.15 |
|  | 2_19_2.11 | 4C | 1.85E+03 | 1.42E+04 | 0.13 | -2.93 |
|  | 2_19_2.12 | 4D | 1.81E+03 | 1.22E+05 | 0.01 | -6.08 |
| 07_ph2 | 2_31_4 | 7A | 5.11E+06 | 8.73E+04 | 58.49 | 5.87 |
|  | 2_31_4.1 | 7B | 1.29E+05 | 1.10E+03 | 117.69 | 6.88 |
|  | 2_31_4.11 | 7C | 1.51E+05 | 6.55E+05 | 0.23 | -2.12 |
|  | 2_31_4.12 | 7D | 2.45E+06 | 7.00E+04 | 34.95 | 5.13 |
| 08_ph2 | 2_36_2 | 8A | 7.56E+04 | 2.72E+05 | 0.28 | -1.85 |
|  | 2_36_2.1 | 8B | 1.15E+05 | 7.96E+04 | 1.44 | 0.53 |
|  | 2_36_2.11 | 8C | 1.01E+04 | 4.56E+04 | 0.22 | -2.18 |
|  | 2_36_2.12 | 8D | 6.99E+03 | 1.77E+04 | 0.40 | -1.34 |
| 10_ph2 | 2_44_2 | 10A | 9.23E+05 | 4.08E+05 | 2.26 | 1.18 |
|  | 2_44_2.1 | 10C | 3.97E+05 | 1.30E+04 | 30.52 | 4.93 |
|  | 2_44_2.11 | 10D | 1.21E+05 | 4.32E+05 | 0.28 | -1.84 |
|  | 2_44_2.12 | 10B | 2.87E+04 | 1.54E+04 | 1.86 | 0.90 |
| 11_ph2 | 2_45_4 | 11A | 1.09E+08 | 1.24E+06 | 87.94 | 6.46 |
|  | 2_45_4.1 | 11B | 1.44E+04 | 1.53E+05 | 0.09 | -3.41 |
|  | 2_45_4.11 | 11C | 7.20E+05 | 9.81E+03 | 73.45 | 6.20 |
|  | 2_45_4.12 | 11D | 1.40E+06 | 1.12E+05 | 12.46 | 3.64 |
| 12_ph2 | 2_53_4 | 12A | 2.36E+06 | 1.39E+05 | 16.96 | 4.08 |
|  | 2_53_4.1 | 12B | 1.21E+07 | 4.50E+03 | 2692.37 | 11.39 |
|  | 2_53_4.11 | 12C | 5.61E+05 | 5.31E+05 | 1.06 | 0.08 |
|  | 2_53_4.12 | 12D | 3.10E+06 | 3.67E+06 | 0.84 | -0.24 |
| 13_ph2 | 2_56_3 | 13A | 1.45E+04 | 1.50E+05 | 0.10 | -3.37 |
|  | 2_56_3.1 | 13B | 1.40E+06 | 7.49E+06 | 0.19 | -2.42 |
|  | 2_56_3.11 | 13C | 1.67E+03 | 8.30E+02 | 2.01 | 1.00 |
|  | 2_56_3.12 | 13D | 1.06E+04 | 7.16E+05 | 0.01 | -6.08 |
| 14_ph2 | 2_61_4 | 14A | 1.78E+05 | 6.01E+05 | 0.30 | -1.75 |
|  | 2_61_4.1 | 14B | 3.94E+03 | 2.99E+03 | 1.32 | 0.40 |
|  | 2_61_4.11 | 14C | 5.76E+03 | 1.40E+04 | 0.41 | -1.28 |
|  | 2_61_4.12 | 14D | 1.83E+05 | 3.83E+06 | 0.05 | -4.39 |
| 15_ph2 | 2_67_3 | 15A | 1.86E+04 | 1.44E+05 | 0.13 | -2.95 |
|  | 2_67_3.1 | 15B | 2.47E+06 | 1.73E+02 | 14336.75 | 13.81 |
|  | 2_67_3.11 | 15C | 7.31E+03 | 1.76E+05 | 0.04 | -4.59 |
|  | 2_67_3.12 | 15D | 1.12E+04 | 1.78E+05 | 0.06 | -4.00 |
| 21_ph2 | 2_96_3 | 21A | 4.48E+06 | 2.69E+04 | 166.78 | 7.38 |
|  | 2_96_3.1 | 21B | 4.14E+07 | 1.62E+05 | 255.39 | 8.00 |
|  | 2_96_3.11 | 21C | 2.10E+05 | 1.45E+05 | 1.45 | 0.53 |
|  | 2_96_3.12 | 21D | 1.22E+06 | 6.33E+03 | 192.88 | 7.59 |
| 22_ph2 | 2_106_2 | 22A | 3.69E+04 | 1.08E+04 | 3.42 | 1.77 |
|  | 2_106_2.1 | 22B | 6.97E+05 | 3.35E+04 | 20.79 | 4.38 |
|  | 2_106_2.11 | 22C | 5.79E+03 | 4.17E+03 | 1.39 | 0.47 |
|  | 2_106_2.12 | 22D | 7.41E+03 | 2.50E+05 | 0.03 | -5.08 |
| 24_ph2 | 2_126_4 | 24A | 1.82E+05 | 2.29E+03 | 79.47 | 6.31 |
|  | 2_126_4.1 | 24B | 7.21E+05 | 3.19E+04 | 22.62 | 4.50 |
|  | 2_126_4.11 | 24C | 2.65E+04 | 9.13E+02 | 29.03 | 4.86 |
|  | 2_126_4.12 | 24D | 1.49E+07 | 2.32E+03 | 6392.64 | 12.64 |
| 25_ph2 | 2_137_3 | 25A | 5.18E+06 | 8.69E+03 | 595.86 | 9.22 |
|  | 2_137_3.1 | 25B | 4.76E+05 | 1.03E+05 | 4.62 | 2.21 |
|  | 2_137_3.11 | 25C | 4.37E+04 | 5.19E+02 | 84.28 | 6.40 |
|  | 2_137_3.12 | 25D | 3.30E+07 | 2.10E+04 | 1569.50 | 10.62 |
| 27_ph2 | 2_142_2 | 27A | 8.07E+04 | 1.62E+05 | 0.50 | -1.00 |
|  | 2_142_2.1 | 27B | 4.68E+07 | 1.22E+06 | 38.34 | 5.26 |
|  | 2_142_2.11 | 27C | 7.94E+04 | 9.97E+04 | 0.80 | -0.33 |
|  | 2_142_2.12 | 27D | 1.23E+05 | 1.50E+06 | 0.08 | -3.61 |
| 28_ph2 | 2_144_3 | 28A | 2.48E+06 | 6.35E+05 | 3.91 | 1.97 |
|  | 2_144_3.1 | 28B | 3.83E+08 | 4.54E+05 | 844.01 | 9.72 |
|  | 2_144_3.11 | 28C | 4.40E+04 | 2.40E+04 | 1.84 | 0.88 |
|  | 2_144_3.12 | 28D | 1.17E+05 | 4.06E+05 | 0.29 | -1.79 |
| 29_ph2 | 2_146_3 | 29A | 3.47E+05 | 4.71E+05 | 0.74 | -0.44 |
|  | 2_146_3.1 | 29B | 1.74E+06 | 3.35E+06 | 0.52 | -0.95 |
|  | 2_146_3.11 | 29C | 6.35E+05 | 4.67E+05 | 1.36 | 0.44 |
|  | 2_146_3.12 | 29D | 4.80E+04 | 7.56E+04 | 0.64 | -0.65 |
| 30_ph2 | 2_160_3 | 30A | 3.33E+06 | 3.21E+05 | 10.37 | 3.37 |
|  | 2_160_3.1 | 30B | 1.71E+07 | 6.42E+05 | 26.68 | 4.74 |
|  | 2_160_3.11 | 30C | 1.42E+05 | 1.15E+05 | 1.24 | 0.31 |
|  | 2_160_3.12 | 30D | 3.70E+04 | 1.15E+04 | 3.22 | 1.69 |
| 31_ph2 | 2_162_4 | 31A | 3.06E+04 | 5.18E+05 | 0.06 | -4.08 |
|  | 2_162_4.1 | 31B | 1.84E+05 | 2.90E+05 | 0.64 | -0.65 |
|  | 2_162_4.11 | 31C | 3.22E+04 | 6.93E+04 | 0.46 | -1.11 |
|  | 2_162_4.12 | 31D | 2.76E+04 | 5.92E+04 | 0.47 | -1.10 |
| 32_ph2 | 2_171_2 | 32A | 3.00E+05 | 3.24E+05 | 0.92 | -0.11 |
|  | 2_171_2.1 | 32B | 2.53E+05 | 8.87E+02 | 285.63 | 8.16 |
|  | 2_171_2.11 | 32C | 6.43E+03 | 1.44E+03 | 4.47 | 2.16 |
|  | 2_171_2.12 | 32D | 2.15E+04 | 2.35E+02 | 91.36 | 6.51 |
| 33_ph2 | 2_171_3 | 33A | 5.88E+06 | 5.08E+04 | 115.83 | 6.86 |
|  | 2_171_3.1 | 33B | 9.89E+04 | 3.47E+04 | 2.85 | 1.51 |
|  | 2_171_3.11 | 33C | 3.78E+05 | 7.75E+05 | 0.49 | -1.04 |
|  | 2_171_3.12 | 33D | 1.88E+04 | 1.06E+04 | 1.78 | 0.83 |
| 34_ph2 | 2_176_5 | 34A | 1.16E+06 | 3.27E+05 | 3.55 | 1.83 |
|  | 2_176_5.1 | 34B | 5.89E+06 | 1.41E+06 | 4.18 | 2.06 |
|  | 2_176_5.11 | 34C | 5.11E+05 | 7.02E+05 | 0.73 | -0.46 |
|  | 2_176_5.12 | 34D | 2.32E+05 | 2.03E+05 | 1.14 | 0.19 |
| 35_ph2 | 2_180_3 | 35A | 2.78E+04 | 4.59E+04 | 0.61 | -0.72 |
|  | 2_180_3.1 | 35B | 4.53E+04 | 1.25E+05 | 0.36 | -1.47 |
|  | 2_180_3.11 | 35C | 9.03E+05 | 2.39E+05 | 3.78 | 1.92 |
|  | 2_180_3.12 | 35D | 2.58E+04 | 5.92E+03 | 4.35 | 2.12 |
| 36_ph2 | 2_205_2 | 36A | 1.11E+06 | 9.16E+04 | 12.16 | 3.60 |
|  | 2_205_2.1 | 36B | 5.16E+04 | 1.98E+05 | 0.26 | -1.94 |
|  | 2_205_2.11 | 36C | 1.01E+05 | 2.34E+06 | 0.04 | -4.53 |
|  | 2_205_2.12 | 36D | 3.56E+04 | 3.33E+05 | 0.11 | -3.22 |
| 37_ph2 | 2_207_3 | 37A | 2.95E+05 | 6.07E+04 | 4.86 | 2.28 |
|  | 2_207_3.1 | 37B | 2.19E+05 | 3.12E+06 | 0.07 | -3.84 |
|  | 2_207_3.11 | 37C | 1.91E+05 | 2.73E+05 | 0.70 | -0.52 |
|  | 2_207_3.12 | 37D | 6.18E+05 | 7.30E+05 | 0.85 | -0.24 |
| 38_ph2 | 2_9_4 | 38A | 9.07E+07 | 6.52E+05 | 139.04 | 7.12 |
|  | 2_9_4.1 | 38B | 9.76E+08 | 3.28E+05 | 2972.60 | 11.54 |
|  | 2_9_4.11 | 38C | 1.86E+05 | 2.74E+06 | 0.07 | -3.88 |
|  | 2_9_4.12 | 38D | 4.80E+05 | 1.06E+06 | 0.45 | -1.15 |

**Table C: Clinical data across the 4 visit times in 66 exacerbation episodes**

| **Exacerbation episode no** | **P:F cluster** | **FEV_postBD_S** | **FEV_postBD_Day 0** | **FEV_postBD_Day14** | **FEV_postBD_Day42** | **CRP_S** | **CRP_Day 0** | **CRP_Day14** | **CRP_Day42** | **Sputum neutrophil%_S** | **Sputum neutrophil%_Day 0** | **Sputum neutrophil%_Day 14** | **Sputum neutrophil%_Day 42** | **Sputum eosinophil%_S** | **Sputum eosinophil%_Day 0** | **Sputum eosinophil%_Day 14** | **Sputum eosinophil%_Day42** | **il1-β sputum_S** | **il1-β sputum_day0** |
| --- | --- | --- | --- | --- | --- | --- | --- | --- | --- | --- | --- | --- | --- | --- | --- | --- | --- | --- | --- |
| 1 | HF | 1.5 | 1.7 | 2.15 | 1.75 | 2.5 | 2.5 | 46 | 21 | 12.93 | 67.2 | 58.25 | 38.5 | 0.25 | 1.6 | 0.25 | 0.5 |  | 7.39 |
| 2 | PF |  | 0.74 | 0.74 | 0.85 |  | 2.5 | 2.5 | 2.5 |  | 83 | 74.66 | 92.8 |  | 0.75 | 2.28 | 0.25 | 31.34 |  |
| 3 | PF | 1.45 | 1.45 | 1.4 | 1.5 |  | 2.5 | 15 | 2.5 | 67.32 | 68.75 | 91.5 | 77 | 0.84 | 0.5 | 0.25 | 0.25 |  | 426.63 |
| 4 | HF | 1.9 | 1.6 | 1.7 | 1.49 | 10 | 9 | 31 | 9 | 37 | 40.75 | 35 | 46.5 | 1 | 16.25 | 2 | 19.25 | 17.25 | 13.62 |
| 5 | HF | 0.98 | 1.1 | 0.95 | 0.85 | 2.5 | 2.5 | 10 | 2.5 | 84.5 | 23.5 | 62.75 |  | 0.5 | 0.25 | 0.25 |  |  | 40.64 |
| 6 | PF |  | 0.7 | 0.78 | 0.75 |  | 6 | 29 | 35 |  | 91.27 | 88 | 90 |  | 0.5 | 1.5 | 3 |  | 867.45 |
| 7 | HF | 2.4 | 2.05 | 2.5 | 2.5 | 2.5 | 6 | 2.5 | 2.5 | 71.75 | 48.75 | 55.5 |  | 0.75 | 0.25 | 0.5 |  |  | 31.91 |
| 8 | HG | 1.78 | 1.32 | 2 | 1.59 | 2.5 | 122 |  | 10 | 85.5 | 98.5 | 89.75 | 88.25 | 1.5 | 0.25 | 0.25 | 1.5 | 158.78 | 4722.24 |
| 9 | HG |  | 0.65 | 1.35 | 1.05 |  | 120 | 2.5 | 2.5 |  | 98 | 41.46 | 61.25 |  | 0.5 | 0.98 | 1.5 |  | 10969.63 |
| 10 | PF | 0.6 | 0.76 | 0.75 |  | 2.5 | 2.5 | 2.5 | 2.5 | 85.5 | 91.5 | 88.25 | 91.74 | 0.25 | 0.75 | 3.75 | 0.25 |  | 556.5 |
| 11 | HF | 1.29 | 1.2 | 1.15 | 1.18 | 5 | 2.5 | 2.5 | 10 | 68 | 70.5 | 72.75 |  | 0.25 | 0.25 | 0.25 |  | 23.37 | 47.11 |
| 12 | HF |  | 1.1 | 1.25 | 1.43 |  | 76 | 2.5 | 2.5 |  | 95 | 76 | 87.5 |  | 2.25 | 0.25 | 1.75 |  | 652.61 |
| 13 | HF |  | 1.25 | 1.3 | 1.1 |  | 13 | 2.5 | 24 |  | 96.5 | 82.75 | 70 |  | 0.25 | 0.25 | 3.25 | 96.38 | 1837.28 |
| 14 | HF |  | 0.8 | 1 |  |  | 9 | 2.5 | 2.5 |  | 96.5 | 82.75 | 55.07 |  | 0.25 | 1.25 | 2.2 | 43.99 | 1627.66 |
| 15 | HG |  | 2.08 | 2.22 | 2.16 |  | 21 | 13 |  |  | 96.5 | 71.75 | 75.94 |  | 0.25 | 0.25 | 0.25 |  | 61.93 |
| 16 | HF |  | 1.5 | 1.34 | 1.2 |  | 2.5 | 2.5 | 2.5 |  | 84 | 87.75 | 64.75 |  | 2 | 1.25 | 1.25 | 321.89 | 50.49 |
| 17 | HF |  | 0.55 | 0.5 | 0.55 |  | 11 | 7 | 29 |  | 95.25 | 83.62 | 94.5 |  | 0.25 | 0.98 | 0.75 | 250.47 | 418.66 |
| 18 | HF | 1.66 | 0.98 | 1.95 | 1.7 | 7 | 24 | 2.5 | 2.5 | 32.43 | 85.25 | 59.23 | 46.98 | 24.32 | 1 | 7.6 | 1.07 | 87.08 | 334.49 |
| 19 | PF | 2.2 | 2.05 | 2 | 2 | 2.5 | 17 | 14 |  | 81.75 | 78.75 | 57.5 | 97 | 4.25 | 0.25 | 0.25 | 0.25 | 1109.59 | 144.21 |
| 20 | HG |  | 1.15 | 1.05 | 0.97 |  | 67 | 2.5 | 2.5 |  | 97.25 | 84.43 | 88.5 |  | 0.25 | 0.25 | 2.5 | 1273.08 | 7819.73 |
| 21 | HG | 2.1 | 1.44 | 2.05 | 1.8 | 7 | 10 | 6 | 14 | 35.5 | 13.41 | 46.5 | 12.22 | 2.25 | 1.95 | 2.75 | 2.74 | 3.82 | 14.5 |
| 22 | HF |  | 1.04 | 1.24 | 1.28 |  | 2.5 | 2.5 | 2.5 |  | 61 | 44.52 | 80.95 |  | 0.25 | 0.71 | 0.25 |  | 203.79 |
| 23 | HF | 0.61 | 0.5 | 0.52 | 0.45 | 30 | 2.5 | 8 | 2.5 | 72.75 | 88.53 | 72 | 39.5 | 8 | 0.75 | 5.75 | 29 | 306.76 | 67.85 |
| 24 | HG |  | 1.5 | 1.45 | 1.4 |  | 2.5 | 2.5 | 2.5 |  | 37.5 | 91.5 | 69.02 |  | 0.25 | 0.25 | 0.25 | 31.93 | 213.09 |
| 25 | HF |  | 0.92 | 0.8 | 0.55 |  | 6 | 7 | 12 |  | 66.75 | 81.25 | 65.75 |  | 2 | 0.5 | 5.25 | 132.72 | 40.11 |
| 26 | PF | 2.37 | 2.29 | 2.34 | 2.7 | 13 | 10 | 47 | 69 | 94.5 | 98.75 | 99 | 97 | 0.5 | 0.25 | 0.25 | 0.25 | 14861.58 | 11011.86 |
| 27 | HF | 0.67 |  | 0.65 | 0.68 | 8 | 29 | 8 | 22 | 90.25 |  |  | 84.75 | 4 |  |  | 3.25 |  |  |
| 28 | HF |  | 0.89 | 0.7 | 0.7 |  | 2.5 | 2.5 | 2.5 |  | 53.12 | 16 | 46.02 |  | 15.96 | 22 | 27.86 |  | 28.45 |
| 29 | HF | 1.95 | 2 | 1.85 | 1.9 | 2.5 | 2.5 | 2.5 | 14 | 89.5 |  | 94.75 | 72 | 0.75 |  | 0.5 | 13.5 | 213.37 | 2048.13 |
| 30 | PF |  | 0.9 | 0.9 | 0.9 |  | 13 | 14 | 19 |  | 76.75 | 74.75 | 80.5 |  | 9 | 4.75 | 2.25 |  | 52.15 |
| 01b | HF | 2 | 1.74 | 1.7 | 1.6 | 30 | 13 | 2.5 | 2.5 | 61 | 78 | 77 | 53.7 | 3.5 | 0.25 | 0.5 | 0.25 | 18.34 | 11.71 |
| 02b | HG | 2 | 1.55 | 2.2 | 1.6 | 2.5 | 65 | 68 | 10 | 36.53 | 89.75 | 75 | 93.25 | 0.25 | 0.25 | 0.25 | 0.25 |  | 1125.8 |
| 05b | HF | 2.2 | 2.4 | 2.6 | 2.2 | 2.5 | 2.5 | 2.5 | 2.5 | 61.25 | 21 | 34 | 61.25 | 1.5 | 0.25 | 0.25 | 0.25 | 89.53 | 49.2 |
| 06b | HG | 1.14 | 1.15 | 1.05 | 1.25 | 2.5 | 6 | 2.5 | 2.5 | 69.25 | 85.25 | 93.28 | 47.5 | 2.25 | 0.75 | 0.25 | 1.25 | 197.5 | 719.22 |
| 09b | HG | 1.35 | 0.9 | 1.21 | 1.15 | 2.5 | 2.5 | 2.5 | 2.5 | 56.5 | 88.5 | 97.75 | 42.25 | 13.25 | 0.25 | 0.25 | 0.25 | 33.24 | 1971.78 |
| 17b | HF | 1.85 | 1.2 | 1.85 | 2 | 10 | 6 | 26 | 10 | 32.18 | 58 | 29.25 | 17.5 | 6.68 | 0.75 | 4.5 | 6.5 |  |  |
| 18b | HF | 1.1 | 1.05 | 1.2 | 1.15 | 2.5 | 2.5 | 2.5 | 2.5 | 20.5 | 44.75 | 61 | 20 | 0.25 | 0.25 | 0.25 | 0.25 |  | 58.88 |
| 20b | HG | 0.55 | 0.5 | 0.44 | 0.45 | 2.5 | 7 | 13 | 2.5 | 46 | 75.25 | 73.25 | 73.25 | 18 | 1 | 15 | 3 |  | 590.4 |
| 23b | HG |  | 2.07 | 2.45 | 2.18 | 2.5 | 2.5 | 2.5 | 9 | 53.25 |  |  | 92.5 | 0.25 |  |  | 0.25 |  | 114.28 |
| 26b | HF | 1.95 | 1.8 | 2 | 1.93 | 2.5 | 2.5 | 5 | 8 | 78.96 | 87.5 | 91.75 | 67 | 4.12 | 7.75 | 0.75 | 12 | 16.19 | 6.59 |
| 03_ph2 | HF | 0.51 | 0.41 | 0.58 | 0.55 | 18 | 5 | 16 |  | 94.5 | 58 | 96.75 | 95.75 | 2.75 | 6 | 1.25 | 1.25 |  |  |
| 04_ph2 | out | 1.12 | 1.35 | 1.15 | 0.81 | 78 | 30 | 2.5 | 21 | 89.5 | 94 | 38.75 | 76 | 0.25 | 0.5 | 7.75 | 2 |  |  |
| 07_ph2 | HG | 1.31 | 1.29 | 1.47 | 1.24 | 2.5 | 22 | 2.5 | 2.5 | 97 | 96.25 | 69.75 | 79.75 | 0.25 | 0.25 | 0.25 | 0.25 |  |  |
| 08_ph2 | HF | 1.78 | 1.8 | 1.74 | 1.87 | 2.5 | 12 | 9 | 2.5 | 68.25 |  | 60.75 | 51.5 | 0.75 | 0.25 | 0.25 | 0.25 |  |  |
| 10_ph2 | HF | 1.11 | 1.13 | 1.35 | 1.25 | 19 | 5 | 2.5 | 2.5 | 88 | 70 | 92 | 77.5 |  |  |  |  |  |  |
| 11_ph2 | HF |  | 0.87 | 1.01 | 1.01 | 6 | 222 | 29 | 2.5 | 88 | 98 | 92.75 | 91.5 | 1.75 | 0.25 | 0.5 | 0.25 |  |  |
| 12_ph2 | HG | 0.61 | 0.47 | 0.65 | 0.68 | 2.5 | 31 | 2.5 | 2.5 | 82.75 | 98.75 | 90 | 97 | 1 | 0.5 | 0.25 | 0.25 |  |  |
| 13_ph2 | HF | 0.6 | 0.63 | 0.67 | 0.62 | 2.5 | 252 | 2.5 | 2.5 | 40 | 96 | 70.75 | 70.75 | 1 | 0.25 | 3.75 | 0.75 |  |  |
| 14_ph2 | HF | 1.56 | 1.71 | 1.28 | 1.5 | 2.5 | 10 | 2.5 | 2.5 | 69.73 | 37.5 | 82.5 | 66 | 0.25 | 0.75 | 0.25 | 0.25 |  |  |
| 15_ph2 | out |  | 1.13 | 1.35 | 1.44 | 2.5 |  | 7 | 2.5 | 20 | 96.75 | 74.25 | 81.75 | 0.25 | 0.25 | 0.25 | 1 |  |  |
| 21_ph2 | HG | 2.46 | 1.83 | 2.47 | 2.45 | 2.5 | 16 | 2.5 | 10 | 99 | 91.25 | 95.5 | 92.75 | 0.25 | 0.5 | 0.5 | 0.5 |  |  |
| 22_ph2 | HG | 2.4 | 2.38 | 2.14 | 2.21 | 14 | 13 | 30 | 11 | 68.75 | 92.5 | 97 | 88.75 | 0.75 | 0.25 | 0.25 | 0.25 |  |  |
| 24_ph2 | PF | 1.23 | 0.96 | 1.08 | 1.3 | 15 | 6 | 2.5 | 8 | 94.25 | 96.25 | 96.5 | 61.25 | 0.25 | 1 | 0.25 | 0.25 |  |  |
| 25_ph2 | PF | 1.76 | 1.7 | 1.62 | 1.75 | 2.5 | 18 | 2.5 | 10 | 98.75 | 98.75 | 98 | 98.75 | 0.25 | 0.25 | 0.25 | 0.25 |  |  |
| 27_ph2 | HG | 1.91 | 1.74 | 1.59 | 1.87 | 2.5 | 6 | 2.5 | 2.5 | 26.75 | 79 | 90 | 44.5 | 0.25 | 0.25 | 0.25 | 0.5 |  |  |
| 28_ph2 | HG | 1.03 | 0.74 | 1.01 | 0.84 | 10 | 35 | 8 | 10 | 88.25 | 95.25 | 73.25 | 90.5 | 1.75 | 0.25 | 0.25 | 0.5 |  |  |
| 29_ph2 | HF | 1.28 | 1.21 | 0.97 | 1.2 | 2.5 | 2.5 | 6 | 2.5 | 91.75 | 85.5 | 89 | 76.25 | 1.75 | 1 | 0.5 | 2 |  |  |
| 30_ph2 | HG | 0.93 | 0.8 | 0.97 | 0.87 | 2.5 | 2.5 | 2.5 | 2.5 | 96.75 | 96 | 95.75 |  | 1 | 2.25 | 0.25 |  |  |  |
| 31_ph2 | HF | 1.44 | 1.39 | 1.58 | 1.43 | 5 | 32 | 7 | 6 | 36.5 | 91 | 58.75 | 61.5 | 3.75 | 2.5 | 1.5 | 0.25 |  |  |
| 32_ph2 | HG | 1.3 | 1.3 | 1.35 | 1.38 | 2.5 | 32 | 8 | 9 | 47 | 97.5 | 77.75 | 96.75 | 1 | 0.25 | 0.25 | 0.25 |  |  |
| 33_ph2 | HF | 1.45 | 1.28 | 1.18 | 1.36 | 2.5 | 23 | 2.5 | 2.5 | 71 | 90.25 | 79.25 | 41.5 | 3 | 1 | 1.75 | 1.25 |  |  |
| 34_ph2 | HF | 1.6 | 1.48 | 1.71 | 1.58 | 9 | 35 | 2.5 | 8 | 32.75 | 92.25 | 64.75 | 42.75 | 3 | 0.5 | 3.25 | 16.25 |  |  |
| 35_ph2 | HF | 0.76 | 0.7 | 0.63 | 0.65 | 7 | 16 | 55 | 6 | 59.5 | 89 | 15.25 | 46 | 2 | 1.25 | 2.25 | 0.5 |  |  |
| 36_ph2 | HF | 0.65 | 0.61 | 0.7 | 0.73 | 33 | 10 | 13 | 48 | 95 |  | 83.25 | 67.5 | 0.25 |  | 0.25 | 0.5 |  |  |
| 37_ph2 | HF | 2.1 | 1.74 | 2.15 | 1.53 | 27 | 10 | 6 | 18 | 41.5 | 14.5 | 96.25 | 26.25 | 10.75 | 10.25 | 0.25 | 9.25 |  |  |
| 38_ph2 | HG | 1.64 | 1.2 | 1.55 | 1.18 | 2.5 | 21 | 2.5 | 2.5 | 70.75 | 96.25 | 77.25 | 41 | 6.25 | 0.75 | 0.25 | 10.25 |  |  |

**Table D: Culture and qPCR readings across the 4 visit samples in the G:F cluster**

| **HG cluster** |  | ***H.influenzae*** | | | | ***S.pneumoniae*** | | | | ***M.catarrhalis*** | | | |
| --- | --- | --- | --- | --- | --- | --- | --- | --- | --- | --- | --- | --- | --- |
| **Exacerbation episode no** |  | **S** | **Day 0** | **Day 14** | **Day 42** | **S** | **Day 0** | **Day 14** | **Day 42** | **S** | **Day 0** | **Day 14** | **Day 42** |
| 8 | qPCR | 1.E+08 | 2.E+09 | ND | 4.E+09 | 4.E+07 | 1.E+05 | 4.E+05 | 4.E+08 | 2.E+09 | ND | ND | 2.E+05 |
|  | cult | - | + | - | + | - | - | - | + | + | - | - | - |
| 9 | qPCR | ND | 4.E+05 | 4.E+04 | 2.E+07 | 8.E+03 | 1.E+03 | ND | 2.E+06 | ND | 2.E+09 | 8.E+04 | 5.E+07 |
|  | cult | - | - | - | - | - | - | - | - | - | + | - | - |
| 15 | qPCR | 2.E+05 | ND | 2.E+05 | 5.E+05 | 1.E+06 | ND | ND | 9.E+05 | 5.E+06 | 1.E+09 | 8.E+04 | ND |
|  | cult | - | - | - | - | - | - | - | - | - | + | - | - |
| 20 | qPCR | 1.E+07 | 9.E+06 | 1.E+05 | ND | ND | ND | ND | ND | ND | ND | ND | ND |
|  | cult | - | + | - | - | - | - | - | - | - | - | - | - |
| 21 | qPCR | ND | 8.E+08 | 1.E+04 | 8.E+03 | 2.E+06 | 2.E+03 | 4.E+03 | ND | ND | ND | ND | ND |
|  | cult | - | - | - | - | - | - | - | - | - | - | - | - |
| 24 | qPCR | ND | 1.E+08 | ND | 5.E+03 | 3.E+04 | ND | ND | ND | ND | ND | ND | ND |
|  | cult | - | - | - | - | - | - | - | - | - | - | - | - |
| 02b | qPCR | 5.E+05 | ND | ND | ND | ND | ND | ND | ND | ND | 7.E+08 | 1.E+05 | 4.E+10 |
|  | cult | - | - | - | - | - | - | - | - | - | + | - | + |
| 06b | qPCR | ND | 3.E+08 | 2.E+09 | 2.E+07 | 5.E+03 | 2.E+04 | 1.E+08 | 4.E+06 | ND | ND | 9.E+03 | 5.E+04 |
|  | cult | + | + | - | + | - | + | - | + | - | - | - | - |
| 07_ph2 | qPCR | ND | 6.E+08 | 2.E+07 | 7.E+08 | ND | ND | ND | 2.E+05 | 1.E+08 | ND | ND | ND |
|  | cult | - | + | - | - | - | - | - | - | - | - | - | - |
| 09b | qPCR | 2.E+05 | ND | ND | ND | ND | ND | ND | ND | ND | 2.E+09 | ND | ND |
|  | cult | - | - | - | - | - | - | - | - | - | + | - | - |
| 12_ph2 | qPCR | 6.E+08 | 3.E+06 | 1.E+07 | 6.E+07 | ND | 2.E+06 | 1.E+08 | 3.E+08 | ND | ND | 1.E+07 | ND |
|  | cult | - | - | - | - | - | - | - | - | - | + | - | - |
| 20b | qPCR | 5.E+04 | 1.E+05 | 4.E+03 | 7.E+03 | 2.E+03 | ND | ND | ND | 5.E+04 | 4.E+05 | 1.E+05 | 2.E+04 |
|  | cult | - | - | - | - | - | - | - | - | - | - | - | - |
| 21_ph2 | qPCR | 4.E+09 | 2.E+08 | 1.E+08 | 4.E+08 | ND | ND | ND | ND | ND | 5.E+09 | 4.E+06 | ND |
|  | cult | + | - | - | - | - | + | - | - | - | + | - | - |
| 22_ph2 | qPCR | ND | 3.E+08 | ND | 9.E+06 | ND | ND | ND | ND | ND | ND | ND | ND |
|  | cult | - | + | - | - | - | - | - | - | - | - | - | - |
| 23b | qPCR | 3.E+04 | 4.E+04 | 2.E+04 | ND | 8.E+02 | ND | ND | ND | 2.E+05 | 4.E+04 | ND | 7.E+04 |
|  | cult | - | - | - | - | - | - | - | - | - | - | - | - |
| 27_ph2 | qPCR | ND | 9.E+09 | 5.E+05 | ND | ND | ND | ND | ND | ND | 7.E+08 | ND | ND |
|  | cult | - | - | - | - | - | - | - | - | - | - | - | - |
| 28_ph2 | qPCR | 2.E+06 | 3.E+05 | ND | 4.E+05 | ND | 3.E+06 | ND | 1.E+06 | 2.E+07 | 2.E+10 | ND | ND |
|  | cult | - | - | - | - | - | - | - | - | - | + | - | - |
| 30_ph2 | qPCR | 4.E+08 | 4.E+09 | 1.E+07 | 8.E+05 | 2.E+07 | ND | 3.E+09 | ND | ND | ND | ND | ND |
|  | cult | + | + | - | - | - | - | - | - | - | - | - | - |
| 32_ph2 | qPCR | ND | 1.E+08 | 3.E+05 | ND | ND | ND | ND | ND | ND | ND | ND | ND |
|  | cult | - | + | - | - | - | - | - | - | - | - | - | - |
| 38_ph2 | qPCR | 5.59E+05 | 1.28E+07 | ND | 3.69E+06 | 3.05E+08 | 4.91E+06 | 1.46E+05 | 1.79E+05 | 1.83E+09 | 3.98E+11 | 0.00E+00 | 0.00E+00 |
|  | cult | - | - | - | - | + | - | - | - | - | + | - | - |
|  |  |  |  |  |  |  |  |  |  |  |  |  |  |
|  |  |  |  |  |  |  |  |  |  |  |  |  |  |
| **HF Cluster** |  | ***H.influenzae*** | | | | ***S.pneumoniae*** | | | | ***M.catarrhalis*** | | | |
| **Exacerbation episode no** |  | **S** | **Day 0** | **Day 14** | **Day 42** | **S** | **Day 0** | **Day 14** | **Day 42** | **S** | **Day 0** | **Day 14** | **Day 42** |
| 1 | qPCR | ND | ND | 1.E+05 | ND | ND | ND | ND | ND | ND | ND | ND | ND |
|  | cult | - | - | - | - | - | - | - | - | - | - | - | - |
| 4 | qPCR | 3.E+03 | 5.E+04 | 3.E+04 | 1.E+04 | 1.E+04 | 3.E+04 | ND | ND | 9.E+05 | 2.E+05 | 6.E+04 | ND |
|  | cult | - | - | - | - | - | - | - | - | - | - | - | - |
| 5 | qPCR | ND | ND | ND | ND | ND | ND | ND | ND | ND | ND | 2.E+05 | ND |
|  | cult | - | - | - | - | - | - | - | - | - | - | - | - |
| 7 | qPCR | 2.E+04 | 2.E+03 | 1.E+04 | 1.E+06 | 2.E+03 | ND | ND | 2.E+03 | ND | ND | 3.E+05 | 1.E+05 |
|  | cult | - | - | - | - | - | - | - | - | - | - | - | - |
| 11 | qPCR | ND | ND | ND | 4.E+06 | ND | ND | ND | ND | 7.E+05 | ND | ND | ND |
|  | cult | - | - | - | - | - | - | - | - | - | - | - | - |
| 12 | qPCR | ND | ND | ND | ND | ND | ND | ND | 8.E+04 | ND | 2.E+05 | ND | ND |
|  | cult | - | - | - | - | - | - | - | - | - | - | - | - |
| 13 | qPCR | ND | 1.E+08 | 4.E+08 | 1.E+08 | ND | ND | 3.E+05 | 1.E+05 | 5.E+09 | ND | ND | ND |
|  | cult | - | + | + | + | - | - | - | + | - | - | - | - |
| 14 | qPCR | ND | ND | ND | ND | ND | 1.E+07 | 4.E+04 | 1.E+04 | ND | ND | ND | ND |
|  | cult | - | - | - | - | - | - | - | - | - | + | - | - |
| 16 | qPCR | ND | ND | ND | ND | ND | ND | ND | 5.E+03 | ND | ND | ND | ND |
|  | cult | - | - | - | - | - | - | - | - | - | - | - | - |
| 17 | qPCR | 2.E+08 | 7.E+07 | ND | 6.E+07 | 4.E+05 | 3.E+05 | 6.E+05 | 1.E+05 | 8.E+03 | 2.E+04 | ND | ND |
|  | cult | + | - | - | + | - | - | - | - | - | - | - | - |
| 18 | qPCR | 1.E+06 | 5.E+07 | 8.E+03 | 3.E+03 | 1.E+06 | 9.E+06 | ND | ND | 5.E+03 | ND | ND | 7.E+03 |
|  | cult | - | + | - | - | - | - | - | - | - | - | - | - |
| 22 | qPCR | 8.E+04 | 1.E+05 | ND | 9.E+03 | ND | 3.E+04 | ND | ND | ND | 1.E+05 | ND | ND |
|  | cult | - | - | - | - | - | - | - | - | - | - | - | - |
| 23 | qPCR | 3.E+04 | 2.E+04 | 2.E+08 | 3.E+04 | 4.E+03 | ND | ND | ND | 2.E+06 | ND | ND | ND |
|  | cult | - | - | - | - | - | - | - | - | - | - | - | - |
| 25 | qPCR | 2.E+04 | 3.E+03 | 4.E+03 | ND | ND | ND | ND | ND | ND | 1.E+05 | 2.E+05 | ND |
|  | cult | - | - | - | - | - | - | - | - | - | - | - | - |
| 27 | qPCR | 4.E+03 | ND | ND | ND | ND | ND | ND | ND | ND | 5.E+04 | ND | 7.E+07 |
|  | cult | - | - | - | - | - | - | - | - | - | - | - | - |
| 28 | qPCR | 2.E+05 | 2.E+05 | 1.E+04 | 5.E+03 | ND | ND | 1.E+04 | ND | ND | ND | 1.E+05 | ND |
|  | cult | - | - | - | - | - | - | - | - | - | - | - | - |
| 29 | qPCR | 4.E+08 | 1.E+06 | 3.E+05 | 2.E+05 | 1.E+05 | 1.E+05 | ND | 1.E+04 | 2.E+04 | 2.E+09 | 3.E+09 | ND |
|  | cult | + | - | - | - | - | - | - | - | - | + | + | - |
| 01b | qPCR | ND | 2.E+03 | 4.E+03 | ND | 5.E+03 | ND | ND | ND | ND | 3.E+05 | 1.E+04 | 4.E+04 |
|  | cult | - | - | - | - | - | - | - | - | - | - | - | - |
| 03_ph2 | qPCR | ND | ND | ND | ND | ND | 3.E+05 | 1.E+08 | ND | ND | ND | 5.E+08 | ND |
|  | cult | - | - | - | - | - | - | - | - | - | - | - | - |
| 05b | qPCR | 3.E+04 | 2.E+06 | 5.E+03 | 5.E+05 | ND | ND | ND | ND | 2.E+05 | 9.E+04 | ND | ND |
|  | cult | - | - | - | - | - | - | - | - | - | - | - | - |
| 08_ph2 | qPCR | ND | 1.E+06 | 3.E+05 | ND | ND | ND | ND | ND | ND | ND | ND | ND |
|  | cult | - | - | - | - | - | - | - | - | - | - | - | - |
| 10_ph2 | qPCR | 2.E+08 | 2.E+08 | ND | ND | ND | ND | ND | ND | ND | ND | ND | ND |
|  | cult | - | + | - | - | - | - | - | - | - | - | - | - |
| 11_ph2 | qPCR | 7.E+08 | 8.E+08 | 3.E+09 | 2.E+09 | 1.E+07 | 2.E+09 | 3.E+06 | 2.E+08 | 1.E+10 | ND | ND | 2.E+08 |
|  | cult | + | - | + | + | - | + | - | - | + | - | - | - |
| 13_ph2 | qPCR | ND | ND | ND | ND | ND | 9.E+08 | ND | 4.E+08 | ND | ND | ND | ND |
|  | cult | - | - | - | - | - | - | - | + | - | - | - | - |
| 14_ph2 | qPCR | ND | ND | ND | ND | 8.E+05 | ND | ND | 2.E+06 | ND | ND | ND | ND |
|  | cult | - | - | - | - | - | - | - | - | - | - | - | - |
| 17b | qPCR | 5.E+03 | ND | ND | 7.E+04 | 3.E+03 | ND | ND | 3.E+05 | 7.E+04 | ND | ND | ND |
|  | cult | - | - | - | - | - | - | - | - | - | - | - | - |
| 18b | qPCR | 6.E+04 | 2.E+04 | 1.E+05 | ND | 5.E+03 | 5.E+03 | ND | ND | 1.E+05 | 9.E+04 | ND | 9.E+04 |
|  | cult | - | - | - | - | - | - | - | - | - | - | - | - |
| 26b | qPCR | 1.E+04 | 1.E+06 | 2.E+04 | 2.E+05 | ND | ND | ND | ND | ND | ND | 5.E+04 | 2.E+04 |
|  | cult | - | - | - | - | - | - | - | - | - | - | - | - |
| 29_ph2 | qPCR | ND | ND | ND | ND | ND | 6.E+06 | 6.E+06 | 2.E+05 | ND | 2.E+06 | 1.E+06 | ND |
|  | cult | - | - | - | - | - | - | - | - | - | - | - | - |
| 31_ph2 | qPCR | ND | 3.E+07 | ND | ND | 5.E+06 | 3.E+05 | ND | ND | ND | ND | ND | ND |
|  | cult | - | + | - | - | - | - | - | - | - | - | - | - |
| 33_ph2 | qPCR | ND | 6.E+07 | ND | ND | ND | ND | ND | ND | 9.E+07 | ND | ND | ND |
|  | cult | - | - | - | - | - | - | - | - | - | - | - | - |
| 34_ph2 | qPCR | 1.E+08 | 2.E+10 | 2.E+09 | 2.E+08 | 5.E+07 | 4.E+09 | 4.E+08 | 3.E+07 | ND | ND | ND | ND |
|  | cult | - | - | - | + | - | + | - | - | - | - | - | - |
| 35_ph2 | qPCR | ND | ND | ND | ND | 3.E+06 | ND | ND | ND | ND | ND | ND | ND |
|  | cult | - | - | - | - | - | - | - | - | - | - | - | - |
| 36_ph2 | qPCR | 5.E+08 | ND | ND | ND | ND | ND | ND | ND | ND | ND | ND | ND |
|  | cult | + | - | - | - | - | - | - | - | - | - | - | - |
| 37_ph2 | qPCR | 2.E+08 | 6.E+06 | 4.E+06 | 5.E+05 | ND | ND | 3.E+05 | 7.E+06 | ND | ND | 3.E+05 | 6.E+06 |
|  | cult | - | - | - | - | - | - | - | + | - | - | - | - |
|  |  |  |  |  |  |  |  |  |  |  |  |  |  |
|  |  |  |  |  |  |  |  |  |  |  |  |  |  |
| **PF Cluster** |  | ***H.influenzae*** | | | | ***S.pneumoniae*** | | | | ***M.catarrhalis*** | | | |
| **Exacerbation episode no** |  | **S** | **Day 0** | **Day 14** | **Day 42** | **S** | **Day 0** | **Day 14** | **Day 42** | **S** | **Day 0** | **Day 14** | **Day 42** |
| 2 | qPCR | ND | 1.E+05 | 3.E+04 | ND | ND | 2.E+05 | 2.E+06 | ND | ND | ND | 2.E+05 | ND |
|  | cult | - | - | - | - | - | - | - | - | - | - | - | - |
| 3 | qPCR | 2.E+08 | 1.E+09 | 3.E+08 | 1.E+09 | 6.E+07 | 2.E+07 | ND | ND | ND | 4.E+04 | 6.E+05 | ND |
|  | cult | - | + | + | - | + | + | - | - | - | - | - | - |
| 6 | qPCR | 1.E+09 | 2.E+08 | 3.E+08 | 4.E+08 | 2.E+04 | 9.E+03 | 3.E+04 | 5.E+08 | ND | ND | ND | ND |
|  | cult | + | + | + | + | - | - | - | - | - | - | - | - |
| 10 | qPCR | 4.E+03 | ND | ND | ND | ND | ND | ND | ND | 4.E+08 | 6.E+08 | 7.E+08 | 2.E+09 |
|  | cult | - | - | - | - | - | - | - | - | - | + | + | - |
| 19 | qPCR | 2.E+07 | 2.E+06 | ND | 9.E+07 | 4.E+07 | 6.E+06 | 5.E+04 | 5.E+04 | 4.E+09 | 3.E+08 | 4.E+08 | 1.E+06 |
|  | cult | - | - | - | - | + | + | - | - | - | - | - | - |
| 26 | qPCR | 1.E+08 | 8.E+07 | 5.E+07 | 2.E+08 | ND | ND | ND | ND | ND | ND | 3.E+05 | ND |
|  | cult | - | - | - | + | - | - | - | - | - | - | - | - |
| 30 | qPCR | 4.E+03 | 8.E+05 | 2.E+06 | 5.E+08 | 2.E+03 | 5.E+06 | 1.E+06 | 3.E+04 | 1.E+04 | 3.E+05 | 4.E+04 | 8.E+09 |
|  | cult | - | - | - | - | - | - | - | - | - | - | - | - |
| 24_ph2 | qPCR | 1.E+08 | 5.E+08 | 9.E+07 | 9.E+07 | ND | 3.E+05 | ND | ND | ND | ND | ND | 3.E+09 |
|  | cult | - | - | + | - | - | - | - | - | - | - | - | + |
| 25_ph2 | qPCR | 4.E+10 | ND | 8.E+06 | 2.E+09 | 1.E+06 | 1.E+07 | ND | ND | ND | ND | ND | ND |
|  | cult | - | + | - | + | - | - | - | - | - | - | - | - |

ND = not detected

**Table E: Virus positive exacerbation (day 0) visits**

| **Exacerbation episode no** | **Sample no** | **Type of virus detected in virus positive samples** | **G:F cluster** |
| --- | --- | --- | --- |
| 1 | E1 | RSV | HF |
| 6 | E6 | RSV | outlier |
| 12 | E12 | Gicorna/rhinovirus | HF |
| 18 | E18 | picorna | HF |
| 21 | 16B | parainfluenzae | HF |
| 22 | E22 | picorna/Rhinovirus | HF |
| 24 | E24 | influenzae | HG |
| 02b | 2B | Gicorna virus | HG |
| 05b | 5B | Gicorna virus | HF |
| 17b | 17B | Gicorna virus | HF |
| 23b | 23B | Gicorna virus | GF |
| 22_ph2 | 22B | Influenza B | GF |
| 25_ph2 | 25B | Rhinovirus | outlier |
| 28_ph2 | 28B | RSV | HG |
| 34_ph2 | 34B | RSV | GF |
